# Targeting PPARγ via SIAH1/2-mediated ubiquitin-proteasomal degradation as a new therapeutic approach in luminal-type bladder cancer

**DOI:** 10.1038/s41419-024-07298-x

**Published:** 2024-12-18

**Authors:** Chih-Chieh Tu, Tsung-Han Hsieh, Cheng-Ying Chu, Yu-Chen Lin, Bo-Jyun Lin, Chun-Han Chen

**Affiliations:** 1 Department of Pharmacology, School of Medicine, College of Medicine, Taipei Medical University, Taipei, Taiwan; 2 Graduate Institute of Medical Sciences, College of Medicine, Taipei Medical University, Taipei, Taiwan; 3 Precision Health Center, Taipei Medical University, Taipei, Taiwan; 4 CRISPR Gene Targeting Core, Taipei Medical University, Taipei, Taiwan; 5 TMU Research Center of Cancer Translational Medicine, Taipei Medical University, Taipei, Taiwan; 6 Cell Physiology and Molecular Image Research Center, Wan Fang Hospital, Taipei Medical University, Taipei, Taiwan

**Keywords:** Apoptosis, Bladder cancer, Proteasome, Preclinical research, Drug development

## Abstract

Bladder cancer (BC) is the second most prevalent genitourinary malignancy worldwide. Despite recent approvals of immune checkpoint inhibitors and targeted therapy for muscle invasive or recurrent BC, options remain limited for patients with non-muscle invasive BC (NMIBC) refractory to Bacillus Calmette-Guérin (BCG) and chemotherapy. NMIBC is more frequently classified as a luminal subtype, in which increased PPARγ activity is a key feature in promoting tumor growth and evasion of immunosurveillance. Cinobufotalin is one of the major compound of bufadienolides, the primary active components of toad venom that has been utilized in the clinical treatment of cancer. We herein focused on cinobufotalin, examining its anticancer activity and molecular mechanisms in luminal-type NMIBC. Our results newly reveal that cinobufotalin strongly suppresses the viability and proliferation of luminal BC cells with minimal cytotoxic effects on normal uroepithelial cells, and exhibits significant antitumor activity in a RT112 xenograft BC model. Mechanistically, our sub-G1-phase cell accumulation, Annexin V staining, caspase-3/8/9 activation, and PARP activation analyses show that cinobufotalin induces apoptosis in luminal-type BC cells. Cinobufotalin significantly inhibited the levels of PPARγ and its downstream targets, as well as lipid droplet formation and free fatty acid levels in RT112 cells. PPARγ overexpression rescued RT112 cells from cinobufotalin-induced apoptosis and mitigated the downregulation of FASN and PLIN4. Finally, we show seemingly for the first time that cinobufotalin promotes SIAH1/2-mediated proteasomal degradation of PPARγ in luminal BC cells. Together, these findings compellingly support the idea that cinobufotalin could be developed as a promising therapeutic agent for treating luminal-type NMIBC.

## Introduction

Bladder cancer (BC) ranks as the second most common genitourinary malignancy worldwide and is clinically categorized as non-muscle-invasive BC (NMIBC) or muscle-invasive BC (MIBC) [1]. Approximately 75% of patients are diagnosed with NMIBC; among them, 50–70% experience disease recurrence, and 10–15% undergo disease progression [2]. Transurethral resection of bladder tumor combined with intravesical Bacillus Calmette-Guérin (BCG) serves as the standard treatment for NMIBC patients. However, it is challenging to manage BCG-refractory patients, and the most common option is lifestyle-altering radical cystectomy [3]. Pembrolizumab, valrubicin, and nadofaragene firadenovec-vncg have been FDA approved for the treatment of BCG-unresponsive NMIBC, but these treatments are not able to provide long-term disease control [4]. Nevertheless, the ongoing worldwide shortage of BCG in many countries warrants the development of alternative therapeutic strategies [5]. Thus, there is a significant unmet need for novel treatments of high-risk NMIBC.

Based on intrinsic molecular subtyping, the majority of BC belongs to the luminal subtype, in which peroxisome proliferator-activated receptor-γ (PPARγ) activity has been identified as a key feature [6]. PPARγ is a member of the nuclear hormone receptor superfamily that plays pivotal roles in regulating adipogenesis, lipid biosynthesis, energy consumption, and storage, inflammation, and differentiation [7]. However, amplification of the *PPARG* gene, and recurrent activating mutations of *PPARγ* and *RXRα* lead to activation of the pro-tumorigenic PPARγ/RXRα pathway in luminal BC [8–10]. PPARγ inverse-agonist treatment or *PPARG* knockout reduced the proliferation, migration, and invasion of BC cell lines harboring *PPARG* genomic amplification or activating mutations of *RXRA* [11]. Meanwhile, long-term usage of thiazolidinediones, the synthetic agonists of PPARγ used to treat type 2 diabetics, is associated with elevated risk of BC development [12, 13]. Given its crucial role in promoting tumorigenesis, PPARγ serves as an attractive therapeutic target for luminal-type BC.

Toad venom is a valuable traditional Chinese medicine obtained from dried secretions of the Asiatic toad (*Bufo gargarizans* Cantor) or black-spectacled toad (*Bufo melanostictus* Schneider). Its liquid extract is extensively utilized for the clinical treatment of several cancer types in China [14]. The primary bioactive components of toad venom are the bufadienolides [15]. Among them, cinobufotalin shows promise as an anticancer agent: it suppresses epithelial-mesenchymal transition via downregulation of β-catenin [16] and reduces lipid synthesis by inhibiting the expression of SREBP1 in liver cancer cells [17]. However, its potency and action mechanisms against BC remain unknown, and further research is needed to support and optimize its clinical applicability in BC.

Here, we newly demonstrate that cinobufotalin suppresses the viability and proliferation of luminal-type BC cells while exerting minimal toxic effects on normal uroepithelial cells in vitro, and displays significant antitumor activity in vivo. Cinobufotalin significantly inhibited the levels of PPARγ and its downstream targets, as well as lipid droplet formation and free fatty acid levels in RT112 cells. PPARγ overexpression rescued RT112 cells from cinobufotalin-induced apoptosis and mitigated the downregulation of FASN and PLIN4. To our knowledge, this is the first study to demonstrate that cinobufotalin promotes apoptosis via SIAH1/2-mediated proteasomal degradation of PPARγ in luminal-type BC cells. Our findings strongly suggest that cinobufotalin could be developed as a promising therapeutic agent for luminal-type BC.

## Materials and methods

### Cell culture and reagents

RT112, UM-UC-3, J82, and SV-HUC-1 cells were purchased from American Type Culture Collection (Manassas, VA, USA). T24, 5637, and RT4 cells were purchased from Bioresource Collection and Research Center (Hsinchu, Taiwan). The passage number of each cell line was below 10, and mycoplasma contamination was routinely tested by PCR analysis. The cells were cultured in RPMI 1640 (RT112 and 5637), McCoy’s 5A (RT4), EMEM (UM-UC-3 and J82), and F12K (SV-HUC-1) growth median at 37 °C with 5% CO_2_, supplemented with 10% FBS and 1X antibiotic-antimycotic (Thermo Fisher Scientific; Waltham, MA, USA). Cinobufotalin (HY-N0880), bortezomib (HY-10227), and menadione (HY-B0332) were purchased from MedChem Express (Monmouth Junction, NJ, USA). Cisplatin (13119) and cycloheximide (14126) were purchased from Cayman Chemicals (Ann Arbor, MI, USA). Other chemicals used in this study were purchased from Sigma-Aldrich (St. Louis, MO, USA).

### MTT and sulforhodamine B (SRB) assay

Cells were seeded into 96-well plates, and then treated with cinobufotalin for 48 h. Cell viability was analysed by incubating with 0.5 mg/mL MTT (3-(4,5-dimethylthiazol-2-yl)-2,5-diphenyltetrazolium bromide) solution at 37 °C with 5% CO_2_ for 1 h, followed by reading the absorbance at 570 nm. The IC_50_ value represents the drug concentration that resulted in a 50% reduction in cell viability. For proliferation analysis, the cells were exposed to cinobufotalin for 48 h. The cells were fixed with 10% TCA, and stained with 0.4% SRB dye, followed by a wash step by 1% acetic acid, and solubilized by 10 mM Tris-base. The absorbance at 515 nm was measured using a spectrophotometer, and the drug concentration causing a 50% inhibition of cell growth (GI_50_) was calculated as previously described [18].

### Flow cytometry analysis

Cells were seeded in six-well plates and treated with cinobufotalin for 48 h. Following treatment, cells were trypsinized, washed with PBS, and fixed at −20 °C with 75% ethanol, and stained with propidium iodide (80 μg/mL) containing 0.1% Triton-X 100 and RNaseA (100 μg/mL) to analyse cell cycle as previously described [18]. To assess apoptosis, cells were collected and stained using the Muse Annexin V & Dead Cell Kit (Luminex, Austin, TX, USA) for 20 min. To analyse mitochondrial membrane potential, cells were stained with the MitoView^TM^ 633 (Biotium, Fremont, CA, USA) for 15 min. Subsequently, the samples were analysed using the Guava Muse Cell Analyzer (Luminex).

### Western blot and immunoprecipitation

Cells were treated with the indicated compounds, and proteins were extracted by using a SDS lysis buffer, separated through SDS-PAGE gels, and transferred onto a polyvinylidene difluoride (PVDF) membrane for subsequent analysis as previously described [18]. The information of primary and secondary antibodies are listed in Supplementary Table S1, and the full length blots are available in Supplementary Materials. The band intensities of each protein were determined by using the Image J software (Version 1.51, National Institutes of Health; Bethesda, MD, USA). For immunoprecipitation, cells were lysed in RIPA buffer on ice for 30 min, and the supernatant was precleaned with protein A/G magnetic beads (88802, Thermo Fisher Scientific) at 4 °C for 2 h, and then incubated with anti-PPARγ antibody (2435, Cell Signaling Technology) at a 1:500 dilution at 4 °C overnight. Finally, the cell lysates were incubated with protein A/G magnetic beads at 4 °C for 2 h, and the beads were washed with RIPA buffer for three times and then subjected to western blotting analysis.

### Plasmid/siRNA transfection and lentiviral shRNA knockdown system

For PPARγ overexpression, the cells were transfected with pcDNA3.1 vector or Flag-tagged PPARγ plasmids (OHu24140D; GenScript, Piscataway, NJ, USA) using TurboFect reagent (Thermo Fisher Scientific) according to the manufacturer’s instructions. For siRNA-mediated knockdown experiments, non-targeting control siRNA pool or ON-TARGET *plus* siRNA pools against human SIAH1 and SIAH2 (Horizon Discovery; Cambridge, UK) were transfected by Lipofectamine RNAiMAX (Thermo Fisher Scientific). For shRNA-mediated knockdown system, lentiviral particles contain shRNA (Supplementary Table S2) were purchased from National RNAi Core Facility (Academia Sinica, Taipei, Taiwan), and stable cells were selected with 2 μg/mL puromycin (InvivoGen, San Diego, CA, USA).

### Reverse transcription quantitative PCR

Treated cell samples had their total RNA extracted using the TRIzol reagent (Thermo Fisher Scientific), and RNA was then reverse transcribed into complementary DNA using the HiScript I TM First Strand cDNA Synthesis Kit from Bionovas (Toronto, ON, Canada). Subsequently, quantitative PCR was conducted using gene-specific primers (Supplementary Table S3) and the RealQ Plus 2× Master Mix Green Kit from Ampliqon (Odense, Denmark).

### Next-generation sequencing (NGS) and bioinformatic analysis

Cells were plated into 6 cm dishes and exposed to cinobufotalin (0.625 μM) for 24 h, and cells were collected by scraping, washed with PBS, and lysed using TRIzol reagent (Thermo Fisher Scientific) to extract RNA. The subsequent steps of library preparation and sequencing were performed as previously described [19]. Differentially expressed genes were identified through DESeq2 (version 1.28.0), and the functional enrichment of Gene Ontology terms and Kyoto Encyclopedia of Genes and Genomes (KEGG) pathways within gene clusters were performed with an R package named clusterProfiler (version 4.0.0) as previously described [19].

### Immunofluorescence and confocal microscopy

5 × 10^4^ cells were plated on coverslips in six-well plates, and exposed to cinobufotalin (0.625 μM) for indicated times. Cells were fixed with 10% formalin for 10 min, permeabilized with 0.1% Triton X-100 for 15 min, and blocked with 2% BSA in PBS for 45 min at room temperature. Then the cells were incubated with primary antibodies at a 1:100 dilution in 0.1% BSA in PBS for 1 h at room temperature, followed by incubation at 4 °C overnight. After incubation for 1 h at room temperature with fluorescence-labeled secondary antibodies (Supplementary Table S1), cells were mounted with mounting medium (23002, Biotium; Fremont, CA, USA) for 24 h at room temperature. Images were acquired with a laser scanning confocal microscope (Stellaris 8; Leica Microsystems, Wetzlar, Germany). Fluorescence intensity was measured using Volocity software (Quorum Technologies, Guelph, ON, Canada), and Pearson’s correlation coefficient was used for quantifying colocalization.

### Oil red O staining and free fatty acid analysis

To stain lipid droplets, the culture medium were removed from the dish and rinse the dish with PBS. Then, fix the cells with 4% paraformaldehyde for 15 min, followed by washing the dish three times with ddH_2_O. Prepare an Oil Red O (ScienCell Research Laboratories, Carlsbad, CA, USA) solution by mixing ddH_2_O and Oil Red O in a 2:3 ratio, filter the solution, and add it to the dish for a 15-min staining. Rinse the dish five times with ddH_2_O, and observe the cells under a microscope. Stained lipid droplets will appear red, and the percentage of area was analysed by Image J (Version 1.51, National Institutes of Health, Bethesda, MD, USA). The free fatty acid level of cells was analysed using the Free Fatty Acid Assay Kit (ab65341; Abcam, Cambridge, UK) following the manufacturer’s instructions.

### In vivo xenograft model

Six-week-old BALB/c nude mice were subcutaneously injected with 1 × 10^6^ RT112 cells. All animal experiments followed ethical standards, and protocols have been reviewed and approved by the institutional Animal Care and Use Committee of Taipei Medical University (IACUC approval no: LAC-2021-0471). Tumor size and mouse body weight were recorded every 2 days. Once the tumors reached approximately 100 mm^3^, mice were grouped into four categories: vehicle, cisplatin 5 mg/kg/i.p./qwk, cinobufotalin 1 mg/kg/i.p./qd, and cinobufotalin 5 mg/kg/i.p./qd. On the 22nd day post-treatment initiation, mice were euthanized. Tumor volume (mm^3^) was calculated using the formula *W*^2^ × *L*/2 (where *W* = width, *L* = length of the tumor in millimeters).

### Statistical analysis

Data are presented as the mean ± SD. The statistical difference was assessed by using the unpaired one tailed Student’s *t*-test in the in vitro assay, and one-way ANOVA was performed to obtain statistical significance in the in vivo assay. The data were considered significant when the *p*-value is lower than 0.05. No statistical methods were employed to predetermine the sample size, and the sample size is indicated in the figure legend. Randomization and blinding were not used.

## Results

### Cinobufotalin differentially inhibits viability and proliferation in luminal BC cell lines and displays potent anti-tumor activity in a RT112 xenograft model of BC

**Fig. 1:**
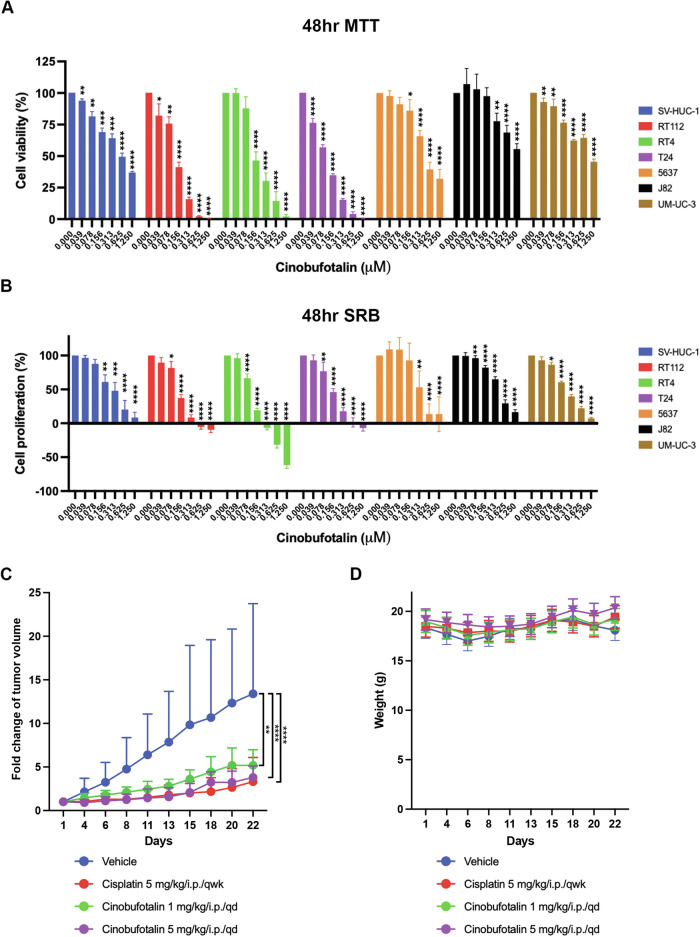
In vitro and in vivo anti-tumor activity of cinobufotalin in BC. BC and normal uroepithelial SV-HUC-1 cells were treated with the indicated concentrations of cinobufotalin for 48 h and cell viability (**A**) and proliferation (**B**) determined using the MTT and SRB assays, respectively. Data are expressed as mean ± SD (*n* = 3) *p < 0.05, **p < 0.01, ***p < 0.001 and ****p < 0.0001 compared with the control group. **C**, **D** Athymic nude mice bearing subcutaneously established RT112 xenograft tumors were randomized into four groups (*n* = 5) and administered the indicated treatments via intraperitoneal injection (i.p.). **C** Tumor sizes were measured every 2 days during treatment with cinobufotalin or cisplatin. Tumor growth curves are depicted as fold changes compared with tumor volume on day 1 and expressed as means ± SD (*n* = 5) **p < 0.01 and ****p < 0.0001 compared with vehicle group. **D** Body weights of mice were measured every 2 days during the drug treatment period. Data are expressed as mean ± SD (*n* = 5).

Although cinobufotalin has been shown to inhibit tumor cell survival in several cancer types [20], preclinical evaluation and molecular mechanisms of action in BC have rarely been reported. We initially assessed the impact of cinobufotalin on cell viability (Fig. 1A) and proliferation (Fig. 1B) in two luminal-type BC cell lines (RT112 and RT4), four basal-type BC cell lines (T24, J82, UM-UC-3, and 5637), and a normal uroepithelial SV-HUC-1 cell line [21]. Cinobufotalin suppressed the viability and proliferation of BC cells at submicromolar concentrations (Table 1), with the highest potency in RT112 and RT4 cells and lowest cytotoxicity in SV-HUC-1 cells. Our results indicate that cinobufotalin exerts selective inhibitory effects on luminal-type BC cells. To evaluate the anticancer efficacy of cinobufotalin in the preclinical setting, we examined its effects in athymic nude mice bearing established RT-112 tumor xenografts. Cinobufotalin induced marked suppression of tumor growth in a dose-dependent manner, exerting anticancer effects comparable to that of cisplatin (Fig. 1C). Notably, mice tolerated all the treatments with no obvious signs of toxicity and displayed no substantial changes in body weight (Fig. 1D), validating the potent antitumor efficacy of cinobufotalin in vivo.

**Table 1:** The IC_50_ and GI_50_ values of cinobufotalin.

Cinobufotalin 48 h							
	SV-HUC-1	RT112	RT4	T24	5637	J82	UM-UC-3
IC_50_ (µM)	0.623	0.121	0.169	0.096	0.616	1.716	0.964
GI_50_ (µM)	0.261	0.121	0.098	0.137	0.383	0.242	0.237
Stage	Normal cells	pTa	pT1/pT2	pTa	–	pT3	pT2–T4
Grade		G2	G1/G1-2	G3	G2	G3	–
Gender		Female	Male	Female	Male	Male	Male
Type		Luminal	Luminal	Basal	Basal	Basal	Basal
		NMIBC	NMIBC	MIBC	MIBC	MIBC	MIBC

### Cinobufotalin induces apoptosis in luminal BC cells

**Fig. 2:**
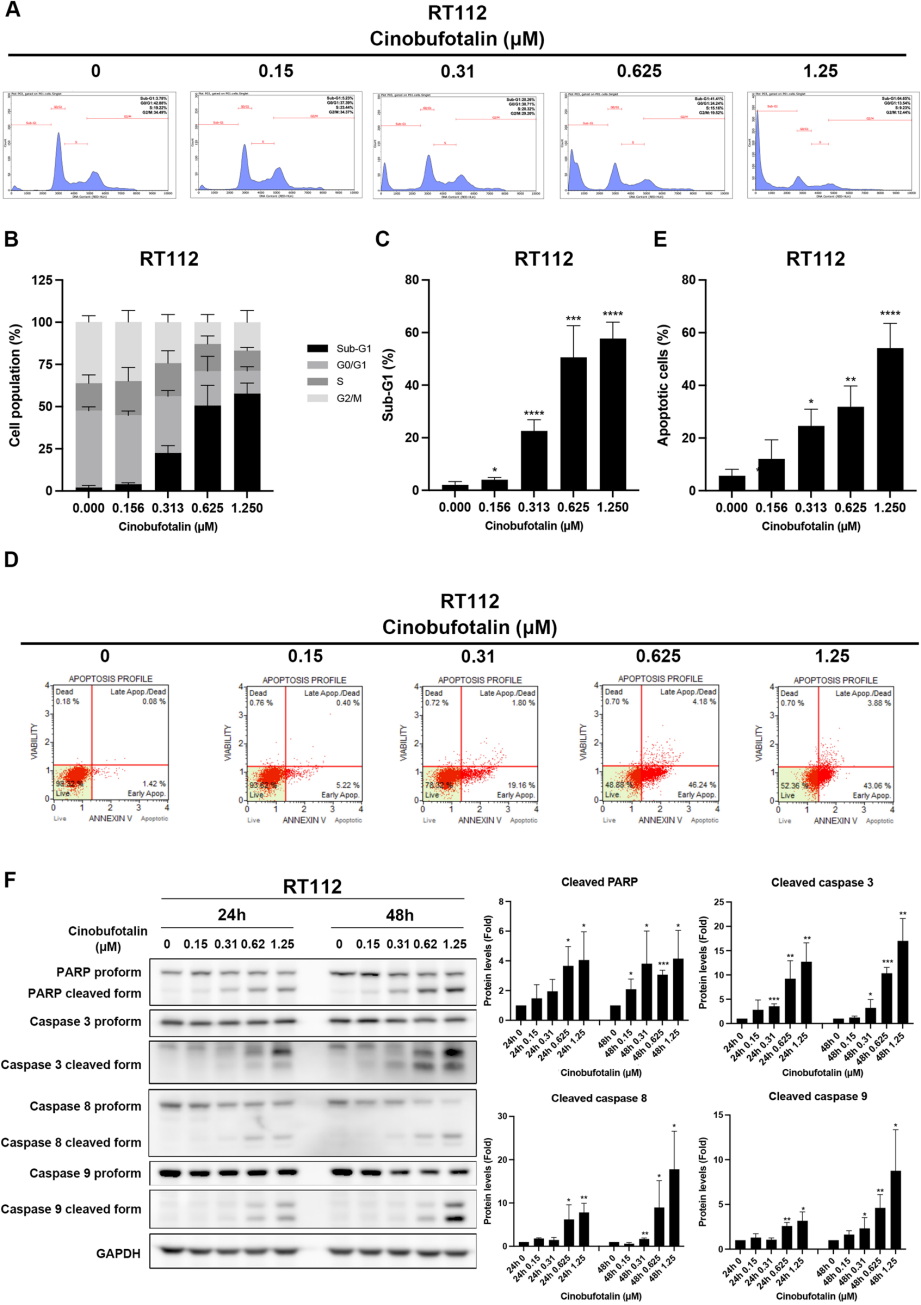
Cinobufotalin promotes cell cycle accumulation at the sub-G1 phase and apoptosis in RT112 cells. **A**–**C** Effects of cinobufotalin on cell cycle distribution in RT112 cells. Cells were treated with different concentrations of cinobufotalin for 48 h and subsequently stained using propidium iodide. The cell cycle was analysed via flow cytometry (**A**). Quantitative data of histograms (**B**, **C**) are expressed as mean ± SD (*n* = 3) *p < 0.05, ***p < 0.001, and ****p < 0.0001 compared with control group. **D**, **E** Effect of cinobufotalin on apoptosis in RT112 cells. Cells were treated with varying concentrations of cinobufotalin for 48 h, stained with Annexin V/7-AAD, and analysed via flow cytometry (**D**). Quantification of early- and late-stage apoptotic cells (**E**) are expressed as mean ± SD (*n* = 3) *p < 0.05, **p < 0.01 and ****p < 0.0001 compared with the control group. **F** RT112 cells were treated with the indicated concentrations of cinobufotalin for 24 h and 48 h and expression of proteins analysed via western blot. Band intensities of each protein were quantified using Image J and normalized to GAPDH. Fold changes compared to control group are expressed as mean ± SD (*n* = 3). *p < 0.05, **p < 0.01 and ***p < 0.001 compared with the control group.

In view of the significant impact of cinobufotalin on luminal BC cells (Fig. 1), RT112 and RT4 were selected for subsequent experiments. The effects of cinobufotalin on cell cycle distribution were further examined via propidium iodide staining. Our results showed extensive accumulation of the sub-G1 population in cinobufotalin-treated RT112 cells (Fig. 2A–C). Annexin V/7-AAD double staining revealed a significant increase in the percentage of early- and late-apoptotic cells in RT112 cells exposed to cinobufotalin (Fig. 2D, E), along with activation of apoptosis-related proteins (PARP, caspase-3/8/9) at both 24 h and 48 h (Fig. 2F). Similar to the above findings, in RT4 cells, treatment with cinobufotalin induced a significant increase in the sub-G1 population (Supplementary Fig. S1A–C), accompanied by activation of apoptotic proteins, such as PARP and caspase-3/8/9, at 24 h and 48 h (Supplementary Fig. S1D). Based on the collective findings, we propose that cinobufotalin promotes the accumulation of cells in the sub-G1 phase and induces apoptosis in luminal BC cells.

### Cinobufotalin downregulates PPARγ signaling pathway in luminal BC cells

**Fig. 3:**
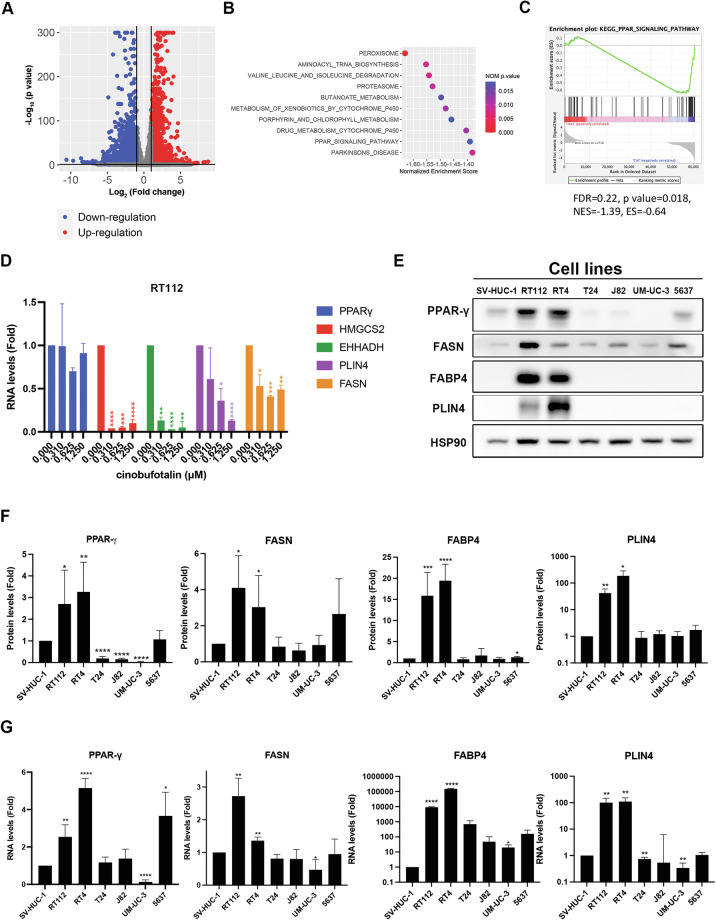
Effect of cinobufotalin on the transcriptome in RT112 cells. **A**–**C** RT112 cells were treated with cinobufotalin (0.625 μM) for 24 h. RNA was extracted using TRIzol reagent, followed by next-generation sequencing (NGS) and bioinformatics analyses (*n* = 2). **A** Volcano plot depicts the differentially expressed genes (*p* adj.<0.05 , |log _2_ (Fold Change)| > 1). Red dots indicate upregulation, while blue dots indicate downregulation. The top 10 KEGG pathways displaying the most significant enrichment in differentially expressed genes (**B**), and the PPAR signaling pathway is significantly enriched (**C**). **D** Effects of cinobufotalin on mRNA levels of PPARγ and its downstream target genes in RT112 cells. Cells were treated with the indicated concentrations of cinobufotalin for 24 h and mRNA expression determined via RT-qPCR. Data are expressed as mean ± SD (*n* = 2). *p < 0.05, **p < 0.01, ***p < 0.001 and ****p < 0.0001 compared with control. **E** Western blot analysis of protein expression of PPARγ and its downstream targets in different BC and normal uroepithelial SV-HUC-1 cells. **F** Band intensities of each protein, determined using Image J software. After normalization to the intensity of HSP90, relative protein levels were determined compared with the SV-HUC-1 group. **G** RT-qPCR analysis of relative mRNA expression levels of PPARγ and its downstream targets in different cell lines compared to normal uroepithelial SV-HUC-1 cells. Data are expressed as mean ± SD (*n* = 3). *p < 0.05, **p < 0.01, ***p < 0.001 and ****p < 0.0001 compared with SV-HUC-1 cells.

To elucidate the mechanisms underlying cinobufotalin-induced apoptosis, next-generation sequencing (NGS) was employed for characterization of differentially expressed genes in RT112 cells. The volcano plot illustrated that 1992 genes were upregulated and 2712 genes were downregulated upon the treatment of cinobufotalin in RT112 cells (Fig. 3A). The results showed that cinobufotalin affects genes associated with metabolism (Fig. 3B) and the PPAR signaling pathway (Fig. 3C, Supplementary Fig. S2A). Given that 91–94% of NMIBC and 47% of MIBC belong to the luminal type, in which PPARγ activity is as a key feature [6], and cinobufotalin displays the highest potency against luminal-type BC cells (Fig. 1), we further examined the effects of cinobufotalin on mRNA levels of PPARγ and its downstream target genes via RT-qPCR analysis. Interestingly, we observed a significant decrease in target genes of PPARγ (*HMGCS2*, *EHHADH*, *PLIN4*, *FASN*) following exposure to cinobufotalin, while the mRNA level of PPARγ itself remained unchanged (Fig. 3D). To validate the importance of PPARγ in luminal-type BC, the PPARγ signaling pathways in different BC and normal uroepithelial SV-HUC-1 cells were compared. The results showed elevated protein (Fig. 3E, F) and mRNA (Fig. 3G) levels of PPARγ and its downstream targets (FASN, PLIN4, FABP4) in the luminal BC cell lines RT112 and RT4. These findings prompted us to further explore the potential role of PPARγ in cinobufotalin-induced apoptosis.

### Cinobufotalin-induced apoptosis is mediated by PPARγ downregulation in luminal BC cells

**Fig. 4:**
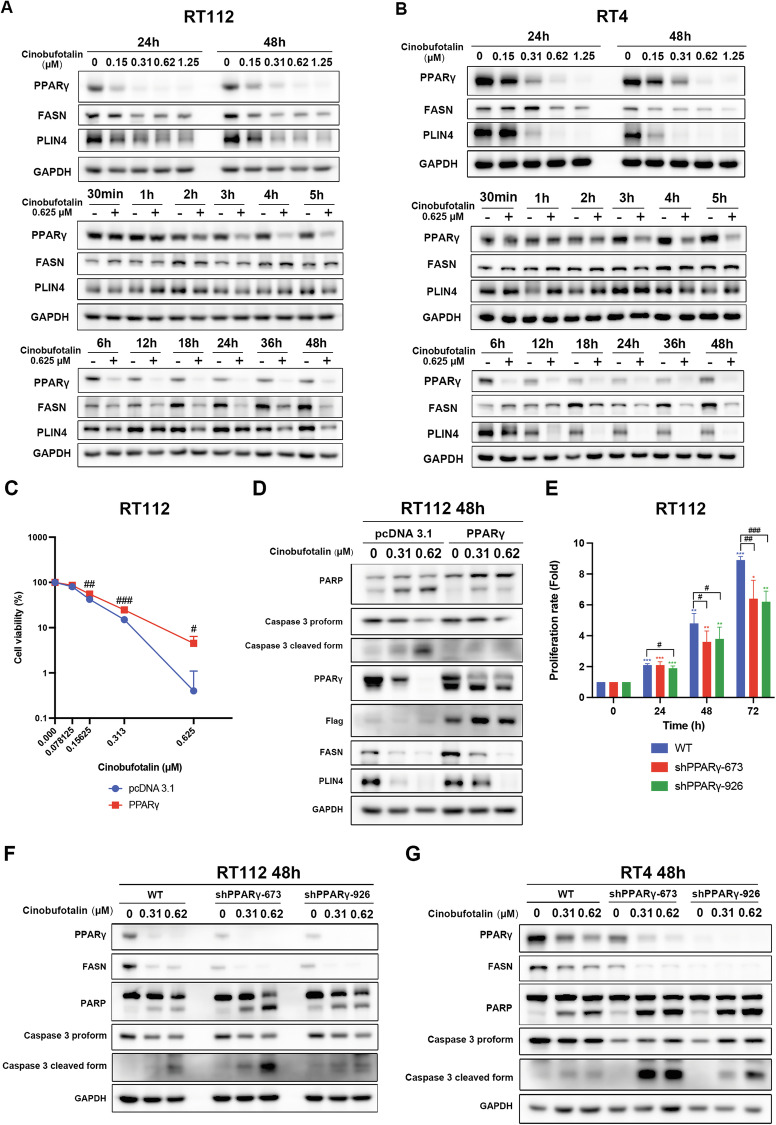
PPARγ downregulation contributes to cinobufotalin-induced apoptosis in luminal BC cells. Cinobufotalin downregulates the protein levels of PPARγ and its downstream targets in luminal BC cells. RT112 (**A**) and RT4 (**B**) cells were treated with different concentrations of cinobufotalin (0.15–1.25 μM) for 24 h and 48 h or 0.625 μM cinobufotalin over a range of time-points (30 min to 48 h) and subjected to western blot analysis. **C** Effect of PPARγ overexpression on cinobufotalin-mediated cytotoxicity in RT112 cells. Cells were transiently transfected with pcDNA 3.1 or PPARγ-Flag plasmids and treated with the indicated concentrations of cinobufotalin for 48 h. Cell viability was determined using the MTT assay. Data are expressed as mean ± SD (*n* = 4) ^**#**^*p* < 0.05, ^**##**^*p* < 0.01, and ^**###**^*p* < 0.001 PPARγ-overexpressing group compared with pcDNA 3.1 group. **D** Overexpression of PPARγ in RT112 cells reverses cinobufotalin-induced apoptosis. Cells were transiently transfected with pcDNA 3.1 or PPARγ-Flag plasmid and treated with the indicated concentrations of cinobufotalin for 48 h. Protein expression was analysed via western blot. **E** Effect of PPARγ knockdown on the proliferation rate of RT112 cells. PPARγ was stably depleted in RT112 cells with two different sequences of shRNA (673, 926), and cell proliferation rates determined using the MTT assay. Data are expressed as mean ± SD (*n* = 4). *p < 0.05, **p < 0.01 and ***p < 0.001 compared with the 0-h group ; ^**#**^*p* < 0.05, ^**##**^*p* < 0.01 and ^**###**^*p* < 0.001 compared with WT group. Knockdown of PPARγ in RT112 (**F**) and RT4 (**G**) cells potentiates cinobufotalin-induced apoptosis. Stable knockdown of PPARγ was achieved with two different sequences of shRNA (673, 926), followed by treatment with cinobufotalin for 48 h. Protein expression was analysed via western blot. Data from statistical analysis of western blots are presented in Supplementary Fig. S7.

To validate the NGS data, western blot analysis was performed, which revealed concentration-dependent downregulation of PPARγ and its downstream targets (FASN and PLIN4) after 24 h and 48 h of treatment of RT112 and RT4 cells with cinobufotalin (Fig. 4A, B; upper panel). Interestingly, cinobufotalin-induced suppression of PPARγ protein was observed as early as 3 h after treatment (Fig. 4A, B; middle panel), while its downstream targets (FASN, PLIN4) were downregulated after 6 h (Fig. 4A, B; lower panel). Given that FASN and PLIN4 play crucial roles in fatty acid synthesis and lipid droplet formation [22], we examined the effects of cinobufotalin on the levels of free fatty acid and lipid droplets by ELISA and Oil Red O staining, respectively. Indeed, cinobufotalin significantly inhibited lipid droplet formation and free fatty acid levels in RT112 cells (Supplementary Fig. S2B–D). PPARγ overexpression rescued RT112 cells from cinobufotalin-induced cytotoxicity (Fig. 4C) and mitigated the downregulation of FASN and PLIN4 as well as activation of apoptotic proteins (PARP, caspase-3) (Fig. 4D). Conversely, shRNA-mediated PPARγ knockdown significantly inhibited RT112 cell proliferation (Fig. 4E), augmented the accumulation of sub-G1 population in cinobufotalin-treated RT112 cells (Supplementary Fig. S3A, B), and potentiated cinobufotalin-induced apoptosis in both RT112 and RT4 cells (Fig. 4F, G). As the PPARγ activity reportedly correlates with the maintenance of mitochondrial membrane potential [23], we further stained the cells with MitoView^TM^ dye to evaluate the mitochondrial integrity by flow cytometry. The data showed that cinobufotalin concentration-dependently suppresses the mitochondrial membrane potential in RT112 cells (Supplementary Fig. S3C, D). Additionally, cinobufotalin significantly decreased the mitochondrial anti-apoptotic protein, MCL1, in RT112 cells (Supplementary Fig. S3E). The collective data strongly suggest that downregulation of PPARγ contributes to cinobufotalin-induced apoptosis in luminal BC cells.

### Cinobufotalin decreases PPARγ expression through the ubiquitin-proteasome system

**Fig. 5:**
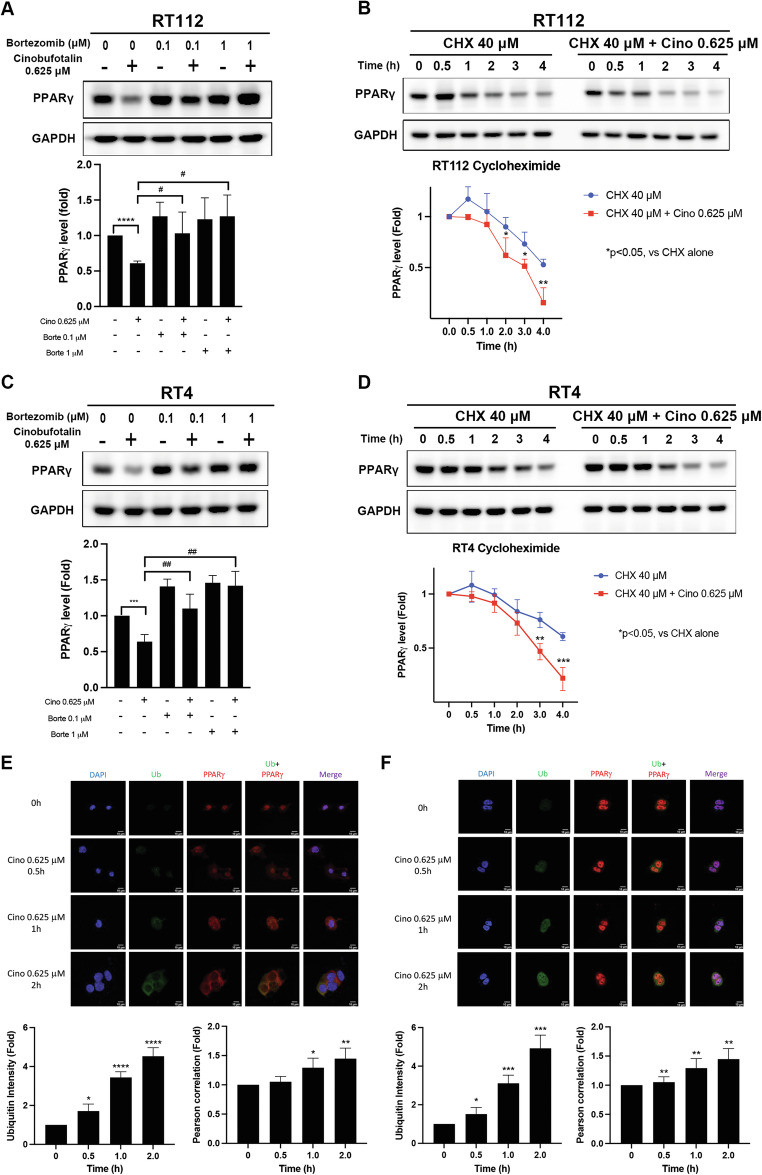
Cinobufotalin promotes the proteasomal degradation of PPARγ in luminal BC cells. **A**–**D** The 20S proteosome inhibitor bortezomib reverses the downregulation of PPARγ induced by cinobufotalin in BC cells. RT112 (**A**) and RT4 (**C**) cells were pretreated with bortezomib (0.1 and 1 μM) for 1 h, followed by cinobufotalin (0.625 μM) for 3 h. Protein levels of PPARγ were analysed via western blot and the band intensities of each protein evaluated using Image J software. Data are expressed as mean ± SD (*n* = 3) ***p < 0.01 and ****p < 0.0001 com*p*ared with control; ^**#**^*p* < 0.05, ^**##**^*p* < 0.01 compared with cinobufotalin alone (0.625 μM). Cinobufotalin decreased the half-life of PPARγ in BC cells. RT112 (**B**) and RT4 (**D**) cells were treated with cycloheximide (40 μM) in the presence or absence of cinobufotalin (0.625 μM) for the indicated times (0.5 h–4 h). Proteins levels were analysed via western blot and the band intensities of each protein determined using Image J software. Data are expressed as mean ± SD (*n* = 3) *p < 0.05, **p < 0.01 and ***p < 0.001 compared with cycloheximide alone. Cinobufotalin promotes ubiquitin-proteasomal degradation of PPARγ in luminal BC cells. RT112 (**E**) and RT4 (**F**) cells were treated with cinobufotalin (0.625 μM) for the indicated times and subjected to immunofluorescence staining and confocal microscopy. Data are expressed as mean ± SD (*n* = 3) *p < 0.05, **p < 0.01, ***p < 0.001, ****p < 0.0001 compared with 0 h group.

Since PPARγ mRNA appears unaffected by cinobufotalin (Fig. 3D), we further investigated whether downregulation of PPARγ could be mediated via post-translational modifications. Notably, treatment with bortezomib, a proteasome inhibitor, significantly reversed the downregulation of PPARγ in RT112 and RT4 cells (Fig. 5A, C). We further used cycloheximide to block protein synthesis, with the aim of determining the protein stability of PPARγ. Our data showed that cinobufotalin significantly shortened the half-life of PPARγ in both cell types (Fig. 5B, D). Given that the ubiquitin-proteasome system is reported to participate in the degradation of PPARγ in adipocytes treated with thiazolidinediones [24], we further examined the effect of cinobufotalin on ubiquitination of PPARγ in RT112 cells via immunocytochemistry and confocal microscopy. Cinobufotalin induced a significant increase in the intensity of ubiquitin staining in a time-dependent manner. Importantly, after 1 h of cinobufotalin treatment, nuclear export of PPARγ and its colocalization with ubiquitin in cytosol were significantly elevated (Fig. 5E). In RT4 cells, cinobufotalin time-dependently increased the intensity of ubiquitin, but its colocalization with PPARγ was instead detected in the nucleus (Fig. 5F). The results suggest that cinobufotalin promotes downregulation of PPARγ through the ubiquitin-proteasomal degradation mechanism.

### SIAH1/2 E3 ubiquitin ligases contribute to cinobufotalin-induced proteasomal degradation of PPARγ in luminal BC cells

**Fig. 6:**
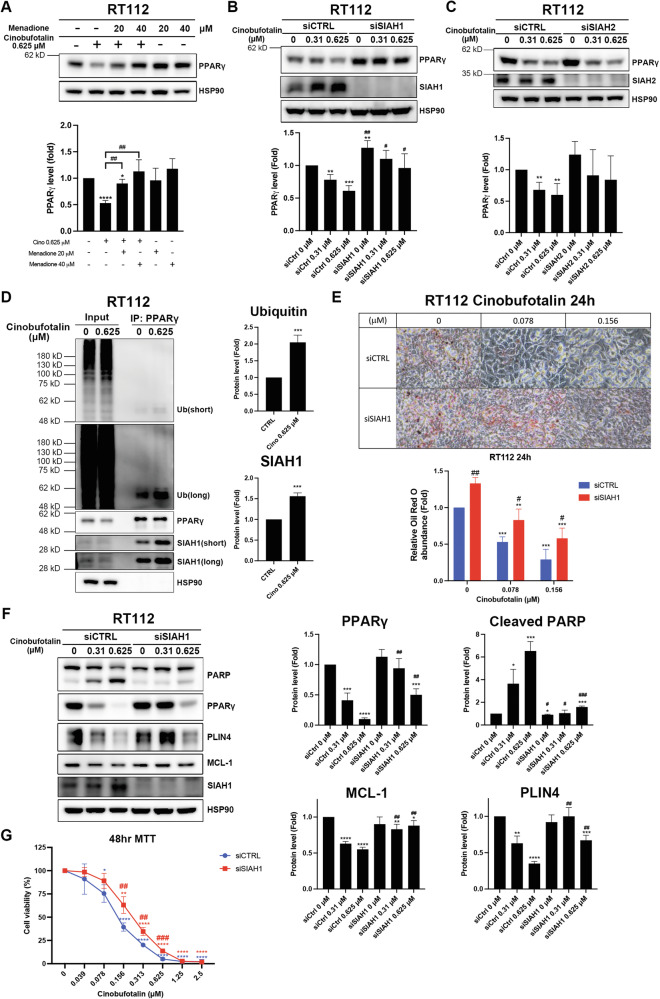
Identification of the E3 ubiquitin ligase mediating cinobufotalin-induced proteasomal degradation of PPARγ in luminal BC cells. **A** The SIAH1/2 inhibitor reverses cinobufotalin-induced degradation of PPARγ in RT112 cells. Cells were pre-incubated with menadione for 30 min, followed by treatment with cinobufotalin for 3 h. Western blot analysis was conducted and the band intensities of each protein determined using Image J software. Data are expressed as mean ± SD (*n* = 3) *p < 0.05 and ****p < 0.0001 compared with control, ^**##**^*p* < 0.01 compared with cinobufotalin 0.625 μM alone. **B**–**D** SIAH1 significantly contributes to the cinobufotalin-induced ubiquitin-proteasomal degradation of PPARγ in RT112 cells. RT112 cells were transfected with non-targeting control (siCTRL), siRNA targeting SIAH1 (**B**) or SIAH2 (**C**), and exposed to the indicated concentrations of cinobufotalin for 3 h. Cells were subjected to western blot analysis and the band intensities of each protein determined using Image J software. Data are expressed as mean ± SD (*n* = 3) **p < 0.01 and ***p < 0.001, compared with control, ^**#**^*p* < 0.05 and ^**##**^*p* < 0.01 compared with the siCTRL group. **D** RT112 cells were exposed to cinobufotalin (0.625 μM) for 2 h and subjected to immunoprecipitation using an antibody against PPARγ, followed by western blot analysis. Band intensities of each protein were determined using Image J software. Data are expressed as mean ± SD (*n* = 3) ***p < 0.001 compared with control (CTRL). **E**, **F** SIAH1 is crucial to cinobufotalin-induced lipid droplet suppression and apoptosis. RT112 cells were transfected with non-targeting control (siCTRL) or siRNA targeting SIAH1 (siSIAH1), and treated with cinobufotalin for 24 h and subjected to Oil Red O staining (**E**). The relative Oil red O-positive areas were analysed with the Image J software. Data are expressed as mean ± SD (*n* = 3) ***p < 0.001 compared with control, ^**#**^*p* < 0.05, ^**##**^*p* < 0.01 compared with the siCTRL group. **F** RT112 cells were transiently transfected with non-targeting control (siCTRL) or siSIAH1. After transfection, the cells were exposed to cinobufotalin for 48 h, and subjected to Western blot analysis. Band intensities of each protein were determined by using the Image J software and normalized to HSP90. Data are expressed as mean ± SD (*n* = 3) *p < 0.05, **p < 0.01, ***p < 0.001 and ****p < 0.0001 compared with control group; ^**#**^*p* < 0.05, ^**##**^*p* < 0.01 and ^**###**^*p* < 0.001 compared with siCTRL group. **G** The effect of SIAH1 knockdown on cinobufotalin-mediated cytotoxicity in RT112 cells. The cells were transiently transfected with control (siCTRL) or SIAH1 siRNA, and treated with indicated concentrations of cinobufotalin for 48 h. Cell viability was assessed using MTT assay. Data are expressed as mean ± SD (*n* = 3) *p < 0.05, **p < 0.01 and ****p < 0.0001 compared to control group; ^**##**^*p* < 0.01 and ^**###**^*p* < 0.001 compared to the siCTRL group.

As E3 ubiquitin ligases play a major role in selective binding and recruiting substrates for ubiquitination [25], we searched the UbiBrowser 2.0 database to identify the E3 ubiquitin ligase participating in cinobufotalin-induced proteasomal degradation of PPARγ [26]. In keeping with previous studies, NEDD4, SIAH2, SMURF1, and VHL were identified as E3 ubiquitin ligases of PPARγ (Supplementary Fig. S4A) [27–30]. Knockdown of *SMURF1*, *VHL*, and *NEDD4* via shRNA had no appreciable reversal effects on degradation of PPARγ (Supplementary Fig. S4B–E). Since human SIAH1 and SIAH2 isoforms share high sequence similarity [31] and both are predicted E3 ubiquitin ligases of PPARγ (Supplementary Fig. S4A), we utilized the SIAH1/2 inhibitor, menadione, to perform rescue experiments [32]. Interestingly, menadione significantly reversed cinobufotalin-induced degradation of PPARγ in both RT112 and RT4 cells (Fig. 6A, Supplementary Fig. S5A). Furthermore, siRNA-mediated elimination of SIAH1 reversed the stimulatory effect of cinobufotalin on PPARγ degradation in RT112 cells, whereas SIAH2 knockdown had no such effect (Fig. 6B, C). Immunoprecipitation experiments revealed that cinobufotalin significantly enhanced PPARγ protein polyubiquitination by detecting endogenous ubiquitin or ectopically expressed HA-tagged ubiquitin in the presence of bortezomib in RT112 cells (Fig. 6D, Supplementary Fig. S5B). Meanwhile, cinobufotalin increased the protein interaction between PPARγ and SIAH1 in RT112 cells (Fig. 6D). Knocking down SIAH1 significantly reversed the effects of cinobufotalin on the inhibition of lipid droplet formation (Fig. 6E), restored protein levels of PPARγ, PLIN4, and MCL-1, and alleviated PARP activation in RT112 cells (Fig. 6F). Meanwhile, SIAH1 knockdown significantly rescued RT112 cells from cinobufotalin-mediated cytotoxicity (Fig. 6G). These data demonstrate that SIAH1 is the ubiquitin E3 ligase that contributes to the proteasomal degradation of PPARγ, which ultimately promotes apoptosis in RT112 cells. In RT4 cells, knockdown of SIAH2, but not SIAH1, rescued degradation of PPARγ in RT4 cells to a significant extent (Supplementary Fig. S5C, D). Immunoprecipitation analysis further confirmed that cinobufotalin increased the ubiquitination of PPARγ and its interactions with SIAH2 in RT4 cells (Supplementary Fig. S5E). Meanwhile, SIAH2 knockdown significantly rescued RT4 cells from cinobufotalin-induced apoptosis (Supplementary Fig. S5F). In view of the collective findings, we suggest that SIAH1/2 E3 ubiquitin ligases mediate cinobufotalin-induced proteasomal degradation of PPARγ in luminal BC cells.

### Subcellular localization of SIAH1 and SIAH2 confers the cell type-specific control of PPARγ degradation in luminal BC cells

**Fig. 7:**
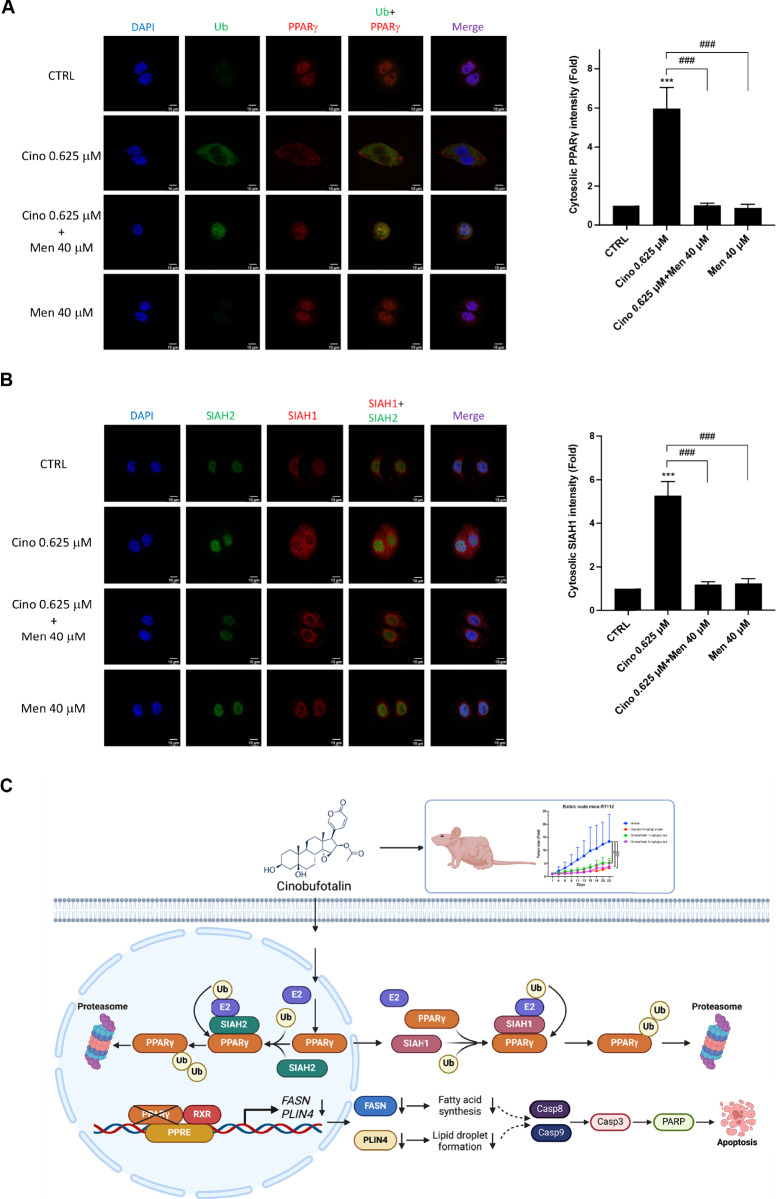
Cytosolic distribution of SIAH1 is crucial to cinobufotalin-induced ubiquitination of PPARγ in RT112 cells. RT112 cells were treated with cinobufotalin (0.625 μM) in the presence or absence of menadione (40 μM) for 2 h and subjected to immunofluorescence staining with antibodies against ubiquitin (Ub) and PPARγ (**A**) or SIAH1 and SIAH2 (**B**). The images were acquired by confocal microscopy, and the data are expressed as mean ± SD (*n* = 3) ***p < 0.001 compared with CRTL group; ^**###**^*p* < 0.001 com*p*ared with cino 0.625 μM group. **C** Proposed anticancer mechanism of cinobufotalin in luminal-type BC cells. Cinobufotalin induces apoptosis by promoting the ubiquitin-proteasomal degradation of PPARγ, facilitated by E3 ligases SIAH1 and SIAH2 in the cytoplasm and nucleus, respectively. The decrease in PPARγ levels leads to the suppression of its downstream targets, FASN and PLIN4, which in turn reduces fatty acid synthesis and lipid droplet formation, potentially activating caspase-dependent apoptosis in luminal BC cells. Furthermore, cinobufotalin exhibits significant antitumor activity in a RT112 xenograft BC model in vivo. PPRE: PPAR response element. The schematic representation was generated using Biorender (^©^BioRender-biorender.com, San Francisco, CA, USA).

SIAH1 and SIAH2 are highly homologous ubiquitin E3 ligases with 90% sequence identity in the substrate-binding domain [32]. However, SIAH1 and SIAH2 contribute to cinobufotalin-induced proteasomal degradation of PPARγ in RT112 and RT4 cells, respectively (Fig. 6B, Supplementary Fig. S5D). To elucidate the underlying mechanism, we utilized immunocytochemistry and confocal microscopy to confirm their subcellular localization in luminal BC cells. In RT112 cells, cinobufotalin significantly increased the cytosolic intensity of PPARγ as well as its colocalization with ubiquitin after 2-h treatment (Fig. 7A). Menadione constrained the distribution of PPARγ and ubiquitin within the nucleus and peri-nuclear region (Fig. 7A). Importantly, cinobufotalin treatment of RT112 cells also increased the cytosolic distribution of SIAH1 without affecting the nuclear expression of SIAH2, and the effect of cinobufotalin on SIAH1 could be reversed by menadione treatment (Fig. 7B). These data suggest that PPARγ is mainly ubiquitinated by SIAH1 in the cytosol of RT112 cells. In RT4 cells, cinobufotalin increased the cytosolic and nuclear intensity of ubiquitin, but its colocalization with PPARγ was only observed in the nucleus (Supplementary Fig. S6A). Menadione decreased the cytosolic distribution of ubiquitin without affecting the nuclear localization of PPARγ (Supplementary Fig. S6B). Importantly, SIAH1 is located in the peri-nuclear region, whereas SIAH2 is mainly observed in the nucleus. The nuclear localization of SIAH2 was not altered by cinobufotalin treatment or the combination with menadione (Supplementary Fig. S6B), suggesting the ubiquitination of PPARγ is mediated by SIAH2 in the nucleus of RT4 cells. In summary, our findings reveal that the subcellular localization is crucial to SIAH1/2-mediated ubiquitination of PPARγ in luminal-type BC cells.

## Discussion

BC ranks as the second most common genitourinary malignancy worldwide [1]. However, therapeutic options remain limited for patients with high-grade NMIBC refractory to BCG and chemotherapy [2] as well as those with MIBC unresponsive to immune checkpoint inhibitors [33]. Cinobufotalin, a member of the bufadienolide family, has attracted significant research interest as a potential anticancer therapy for various tumor types, including hepatocellular and lung cancer [16, 17, 34]. However, the efficacy and pharmacological mechanisms of action of cinobufotalin in BC remain to be established. The current study revealed that cinobufotalin exhibits differential cytotoxicity towards luminal BC cells at submicromolar concentrations while exerting minimal toxicity in normal uroepithelial cells. Moreover, the antitumor activity of cinobufotalin was comparable to that of cisplatin in vivo. To our knowledge, this is the first study to demonstrate that cinobufotalin induces apoptosis by promoting the ubiquitin-proteasomal degradation of PPARγ, facilitated by E3 ligases SIAH1 and SIAH2 in the cytoplasm and nucleus, respectively. The decrease in PPARγ levels leads to the suppression of its downstream targets, FASN and PLIN4, which in turn reduces fatty acid synthesis and lipid droplet formation, potentially activating caspase-dependent apoptosis in luminal BC cells (Fig. 7C). Cinobufotalin has been proposed as an SREBP1 inhibitor based on molecular docking analysis and thermal stabilization assay, but the evidence of direct binding between cinobufotalin and purified SREBP1 protein through plasmon resonance analysis was not provided [17]. However, the data show that cinobufotalin suppresses SREBP1 expression and its interaction with sterol regulatory elements, significantly reducing lipogenesis in hepatocellular carcinoma cells [17]. These findings further strengthen our observations that cinobufotalin targets lipid metabolism by promoting the ubiquitin-proteasomal degradation of PPARγ. Whether cinobufotalin affects SREBP1 in luminal BC cells is worthy to study in the future. Therefore, cinobufotalin is a highly promising candidate for the treatment of luminal-type BC, which could eventually be utilized for the development of novel therapeutic alternatives in the future.

Based on intrinsic molecular subtyping, approximately 91–94% of NMIBC and 47% of MIBC belong to the luminal subtype, in which PPARγ activity is a key feature [6]. Amplification of the *PPARG* gene, and recurrent activating mutations of PPARγ and RXRα that lead to activation of PPARγ/RXRα pathway PPARγ have been linked to a pro-tumorigenic role of PPARγ in luminal-type BC [8–10]. In contrast, other studies suggest that PPARγ agonizts inhibit cancer cell proliferation in lung adenocarcinoma and hepatocellular carcinoma [35, 36]. The conflicting roles of PPARγ agonists in regulating cancer cell proliferation may result from the off-target effects of thiazolidinediones [37, 38]. Additionally, long-term usage of thiazolidinediones has been associated with elevated risk of BC development [12, 13]. Therefore, PPARγ is considered as an attractive therapeutic target for luminal-type BC. In this investigation, cinobufotalin showed the highest potency in luminal BC cells (Fig. 1) with higher levels of PPARγ and downstream target genes relative to other BC cell types (Fig. 3E–F). Recurrent activating mutations of PPARγ and RXRα have been linked to pro-tumorigenic PPARγ/RXRα pathway activation in luminal bladder tumors [8]. Meanwhile, long-term usage of thiazolidinediones, the synthetic agonists of PPARγ used to treat type 2 diabetics, is associated with elevated risk of BC development [12, 13]. PPARγ^High^/RXRα^S427F/Y^ has been shown to impair the secretion of inflammatory cytokines and CD8^+^ T-cell infiltration and confer partial resistance to immunotherapy in both clinical datasets and animal tumor models [39]. Given the significant roles of PPARγ in tumorigenesis and immune evasion in luminal BC, future research should focus on the impact of cinobufotalin on immune surveillance.

In this study, SIAH1 and SIAH2 were identified as the E3 ligases responsible for cinobufotalin-induced proteasomal degradation of PPARγ in luminal BC cells (Fig. 6, Supplementary Fig. S5). SIAH1 and SIAH2 are highly homologous and functionally redundant ubiquitin E3 ligases implicated in the regulation of metabolism and the hypoxic response [40]. PPARγ ubiquitination and ligand-dependent activation are promoted by SIAH2, which is increased during adipogenesis [28]. Here, the SIAH1/2 inhibitor menadione significantly prevented the degradation of PPARγ following cinobufotalin treatment (Fig. 6A, Supplementary Fig. S5A). Interestingly, knockdown of SIAH1 and SIAH2 reversed the cinobufotalin-mediated degradation of PPARγ in both RT112 and RT4 cells, respectively (Fig. 6B, Supplementary Fig. S5D). Immunocytochemistry and confocal microscopy data suggest that PPARγ is mainly ubiquitinated by SIAH1 in the cytosol of RT112 cells, whereas the ubiquitination of PPARγ is mediated by SIAH2 in the nucleus of RT4 cells (Fig. 7, Supplementary Fig. S6). In luminal BC cells, SIAH1 and SIAH2 exhibit distinct subcellular localization patterns, with SIAH1 primarily located in the peri-nuclear region and SIAH2 within the nucleus (Fig. 7B, Supplementary Fig. S6B). The nuclear expression of SIAH1 has been associated with its oncoprotein properties in liver and breast cancers, although the mechanism behind its nuclear localization remains unclear [41, 42]. Phosphorylation of SIAH2 by p38 promotes its nuclear export and enhances its E3 ligase activity, leading to the proteasomal degradation of PHD3 and consequently stabilizing HIF1α under hypoxia conditions [43]. However, owing to the high similarity between SIAH1 and SIAH2 and lack of specific inhibitors, we were unable to ascertain whether these two proteins work in concert or sequentially in luminal BC cells. Further research is therefore warranted to elucidate the upstream regulators of cinobufotalin-induced ubiquitin-proteasomal degradation of PPARγ.

In conclusion, data from the current study suggest that cinobufotalin displays selective cytotoxic activity against luminal BC cells in vitro and significant antitumor activity in vivo. In terms of its mechanism of action, cinobufotalin triggers apoptosis through SIAH1/2-dependent proteasomal degradation of PPARγ. These findings provide compelling evidence supporting the utility of cinobufotalin as a novel therapeutic agent, highlighting the need for further research on the upstream regulation of PPARγ degradation and its potential application in modulating anticancer immunity in luminal BC.

## Supplementary information

Original data

Supplementary materials

## Data Availability

All data generated or analysed during this study are available from the corresponding author on reasonable request.
