# Supplementary material for: Targeting PPARγ via SIAH1/2-mediated ubiquitin-proteasomal degradation as a new therapeutic approach in luminal-type bladder cancer
Source: Cell Death Dis. 2024 Dec 18;15(12):908. doi: 10.1038/s41419-024-07298-x (PMC11655661; doi:10.1038/s41419-024-07298-x)
Supplement: Supplementary file 1 — Original data [file 41419_2024_7298_MOESM1_ESM.pdf]

Original data of Western blots

Fig. 2F

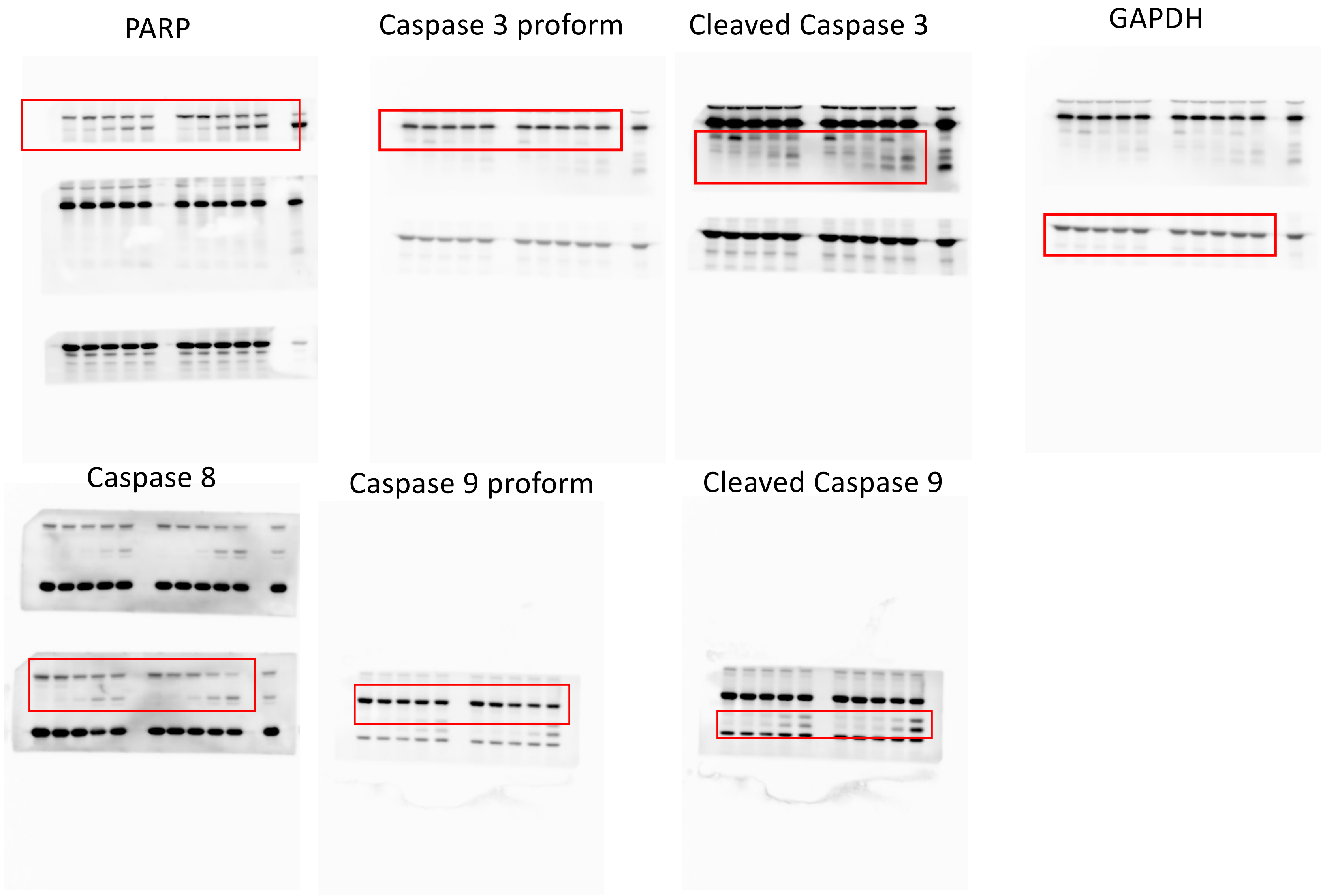
PARP
Caspase 3 proform
Cleaved Caspase 3
GAPDH
Caspase 8
Caspase 9 proform
Cleaved Caspase 9

Fig. 3E

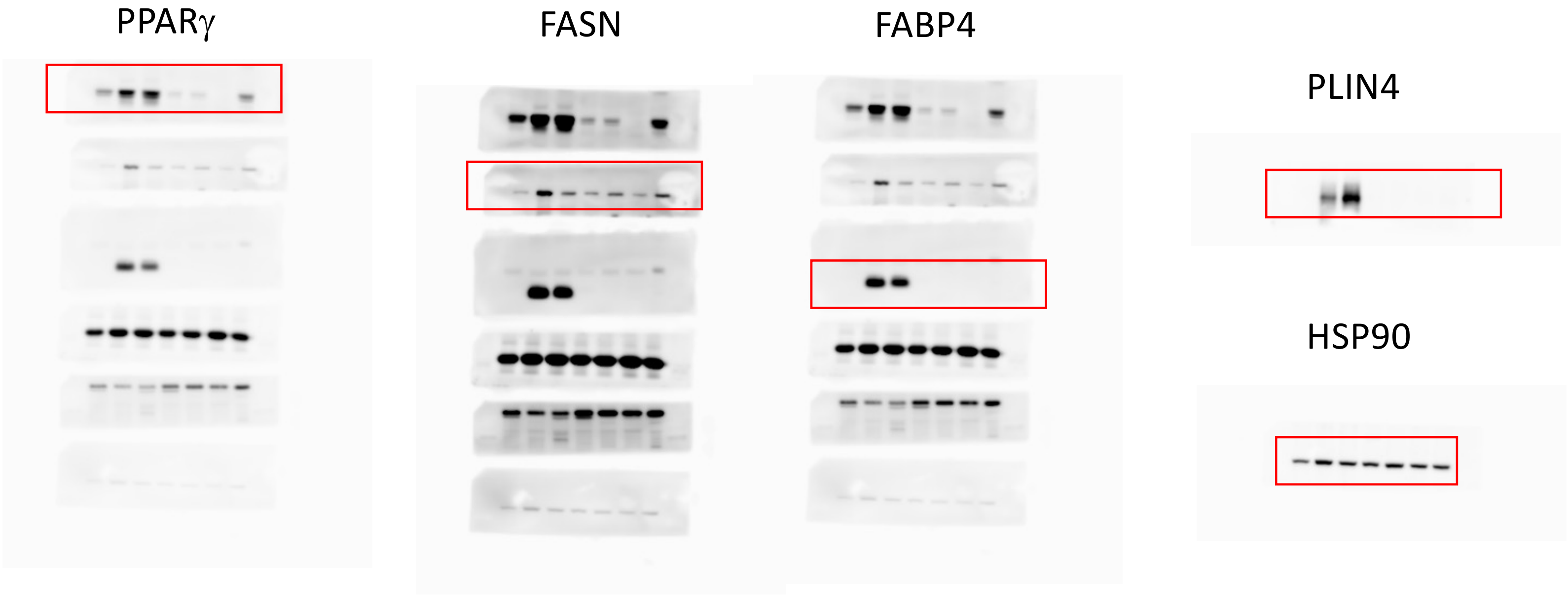

Fig. 4A (Upper)

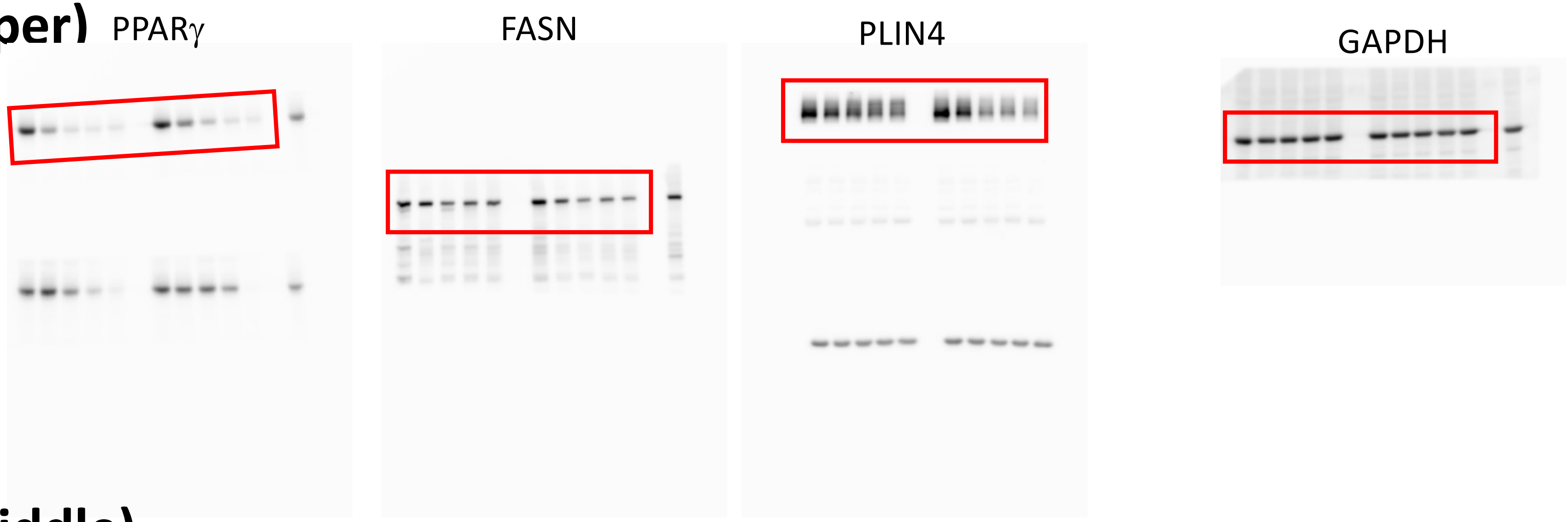

Fig. 4A (Middle)

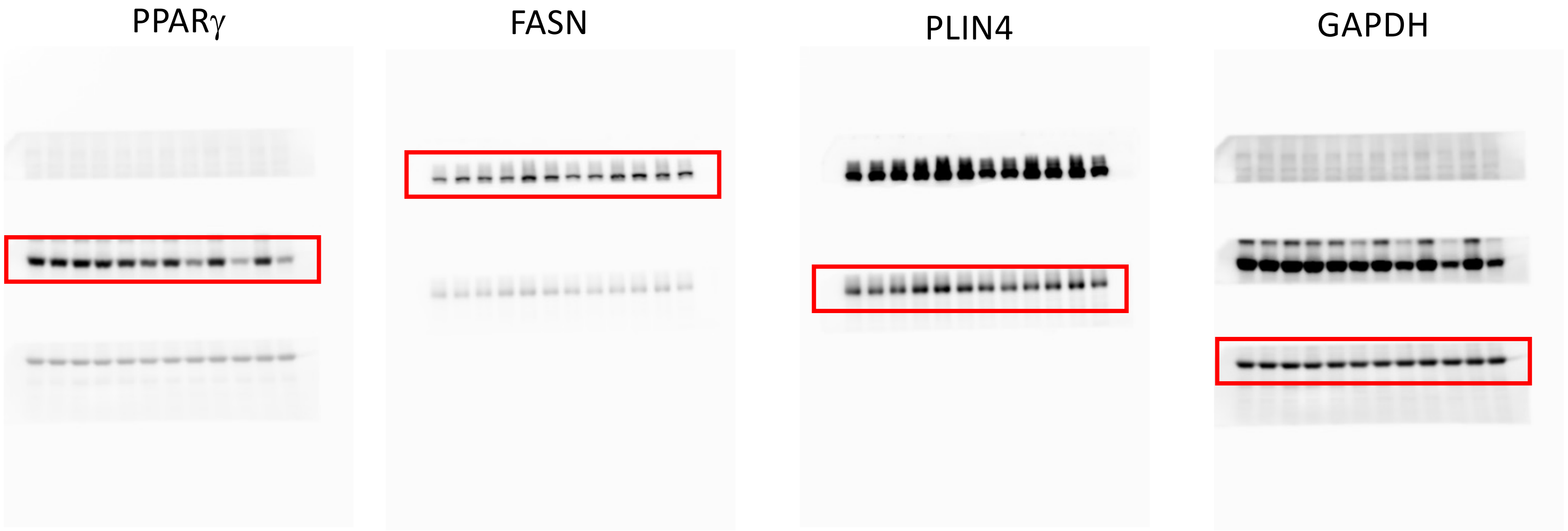

Fig. 4A (Lower)

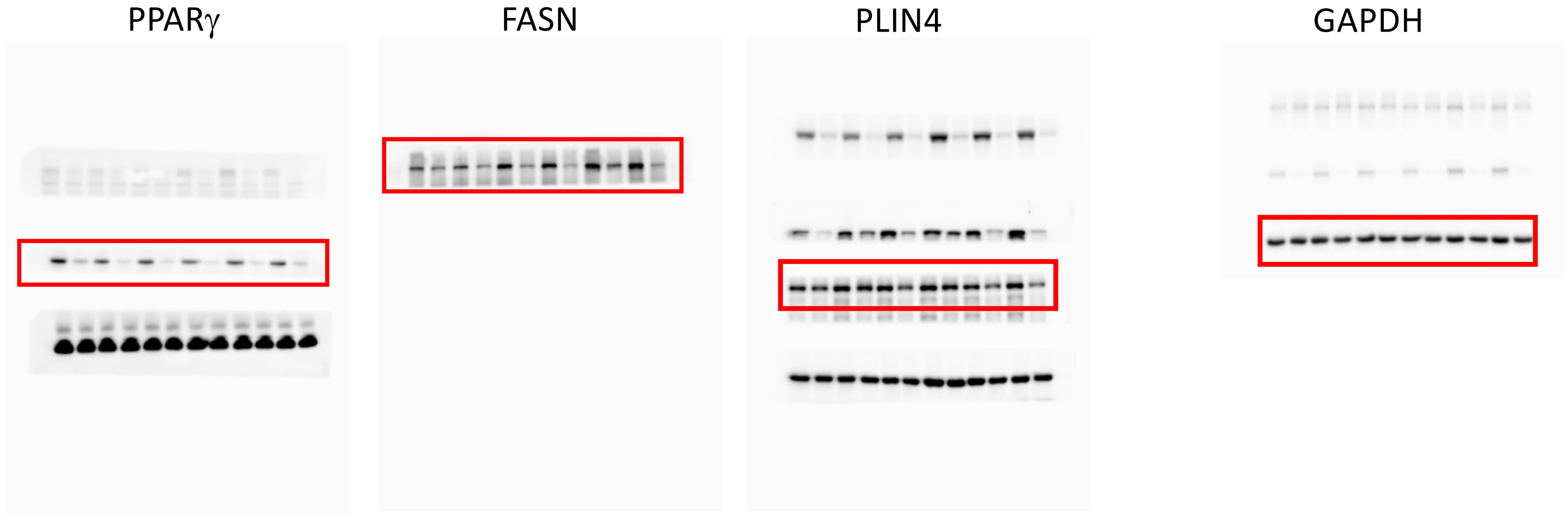

Fig. 4B (Upper)

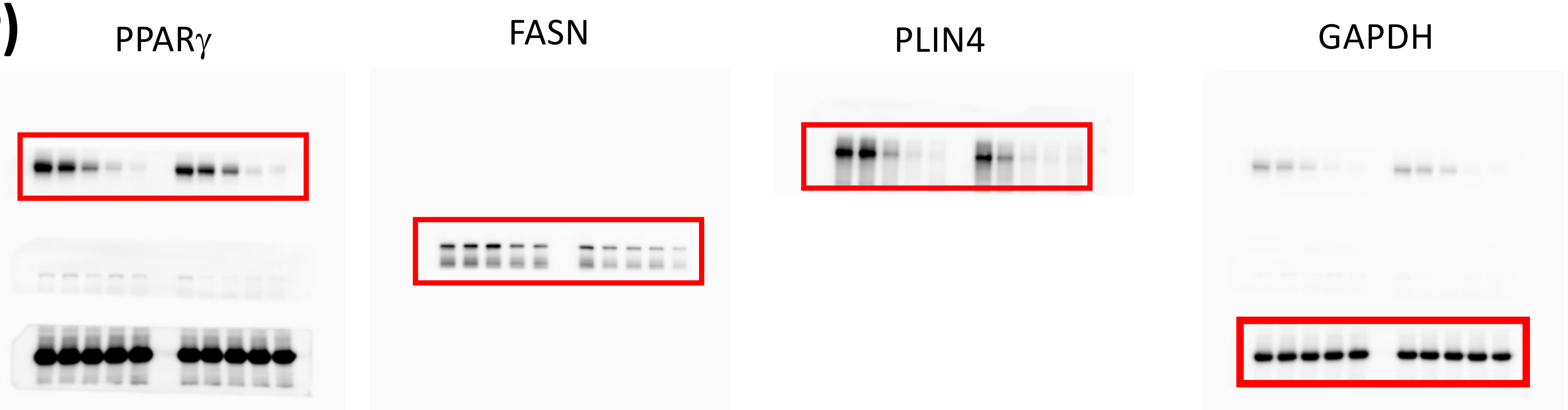

Fig. 4B (Middle)

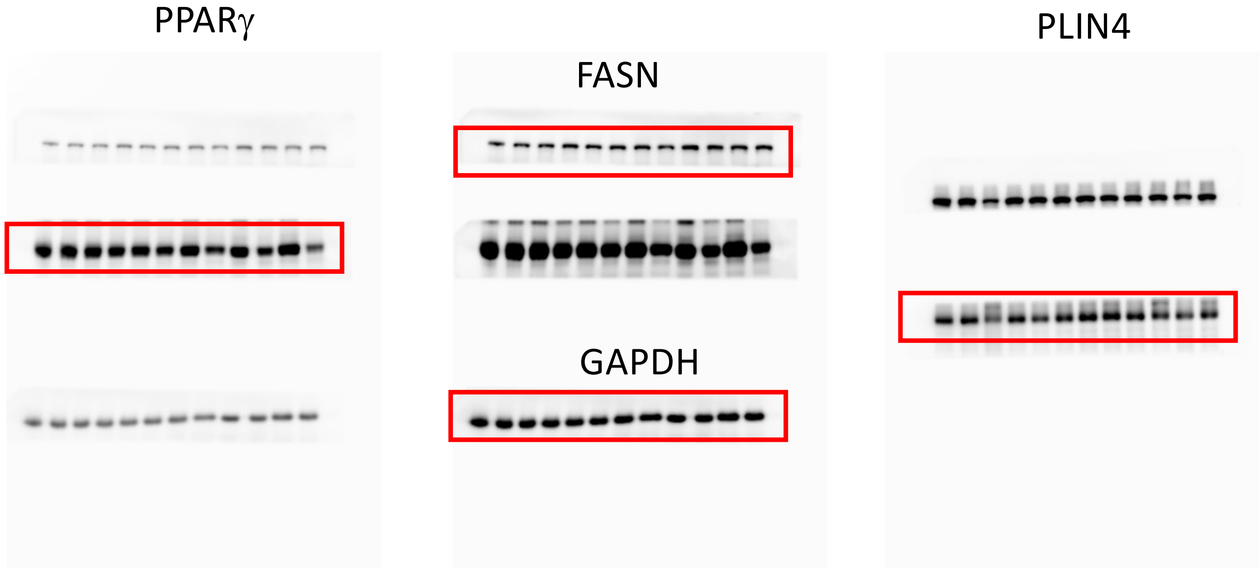

Fig. 4B (Lower)

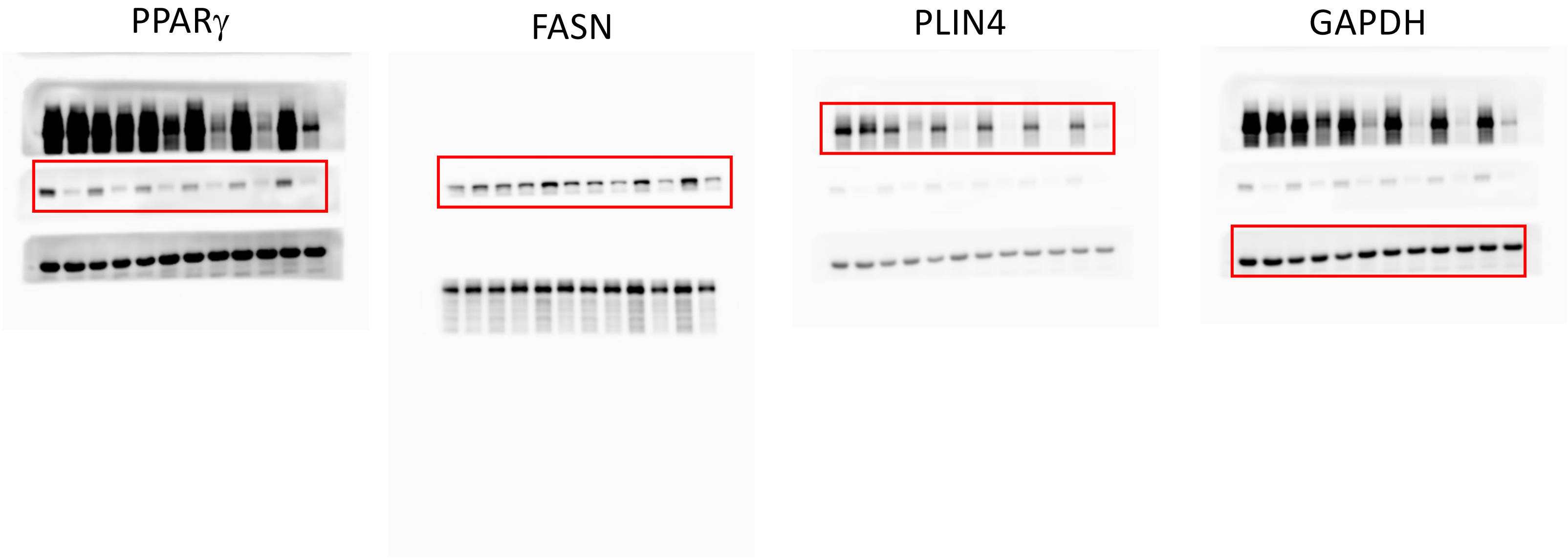

Fig. 4D

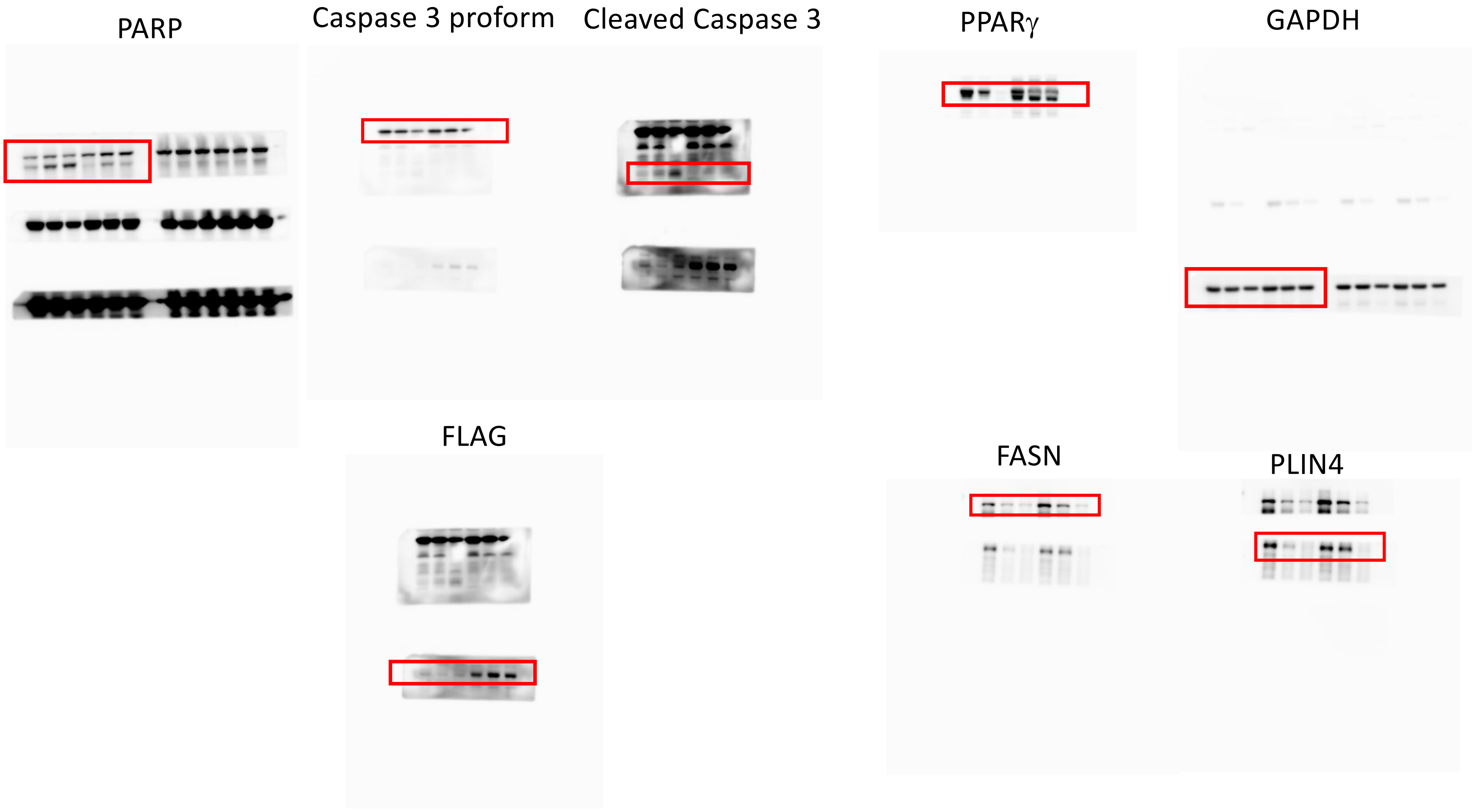
PARP
Caspase 3 proform
Cleaved Caspase 3
PPARγ
GAPDH
FLAG
FASN
PLIN4

Fig. 4F

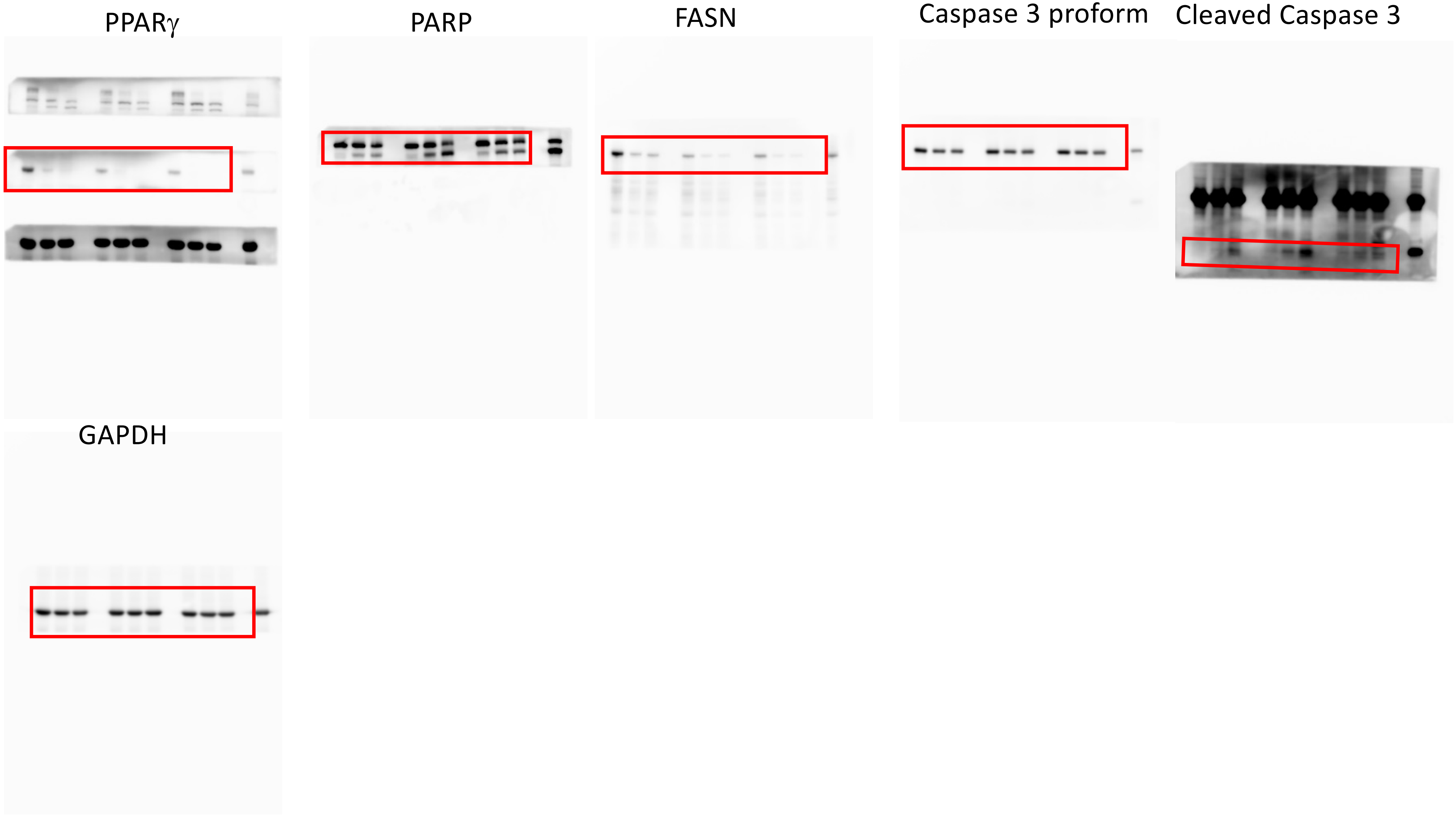

Fig. 4G

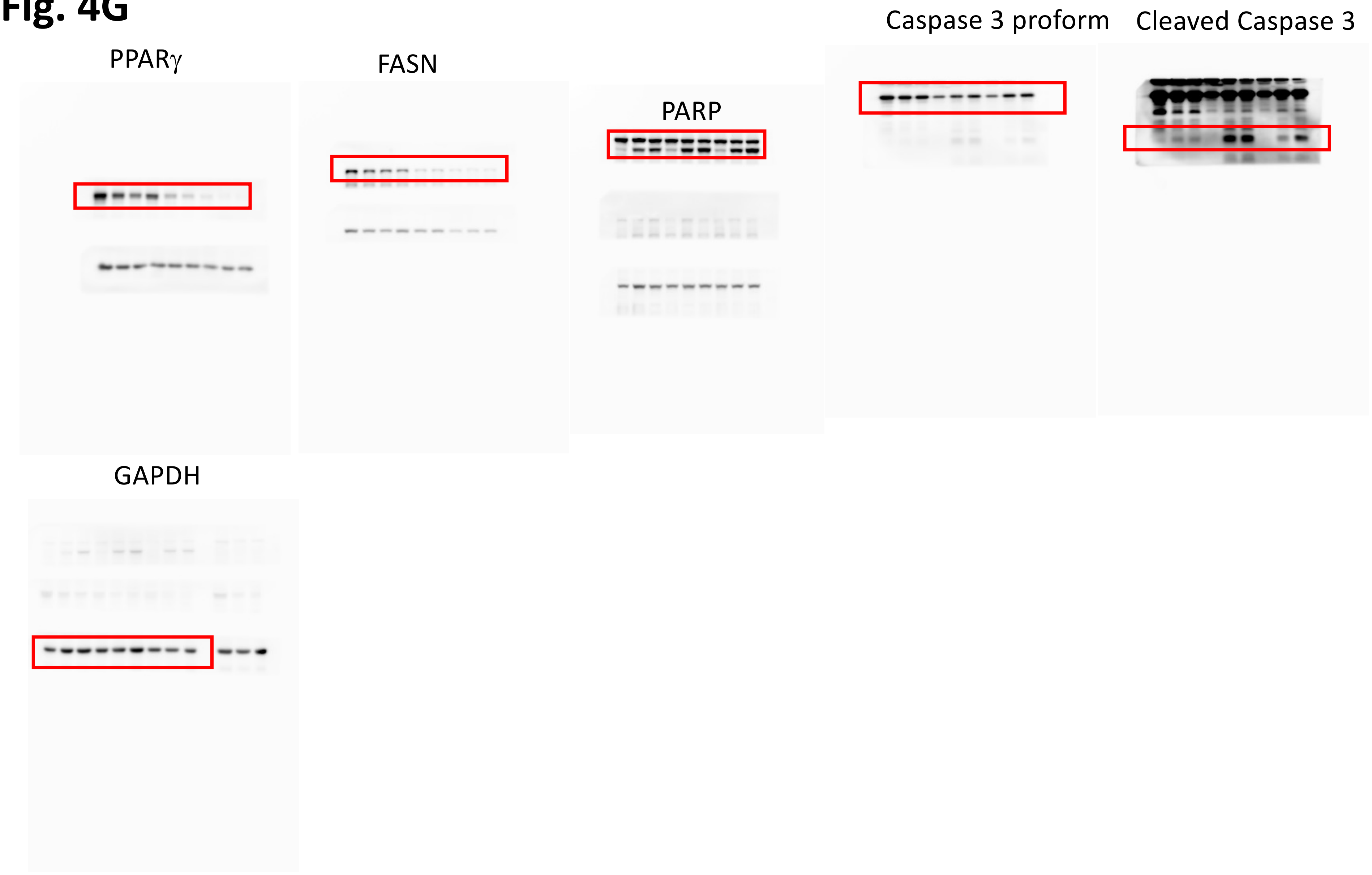

Fig. 5A

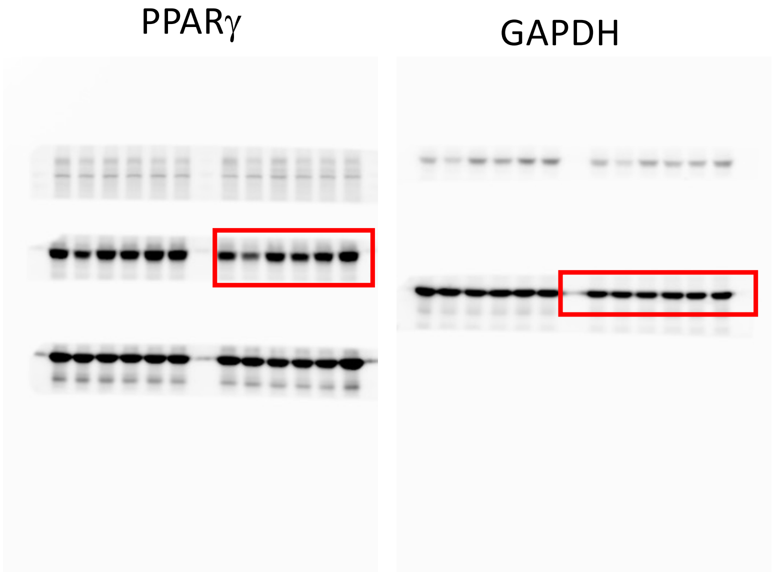

Fig. 5B

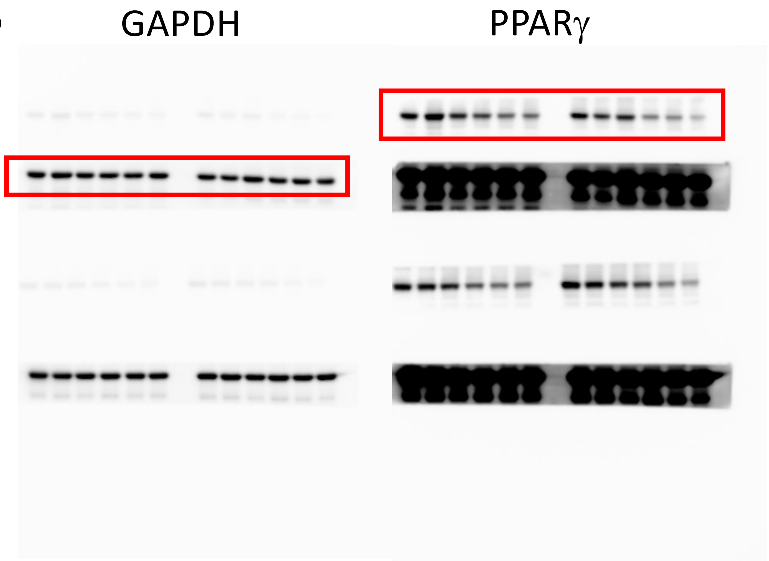

Fig. 5C

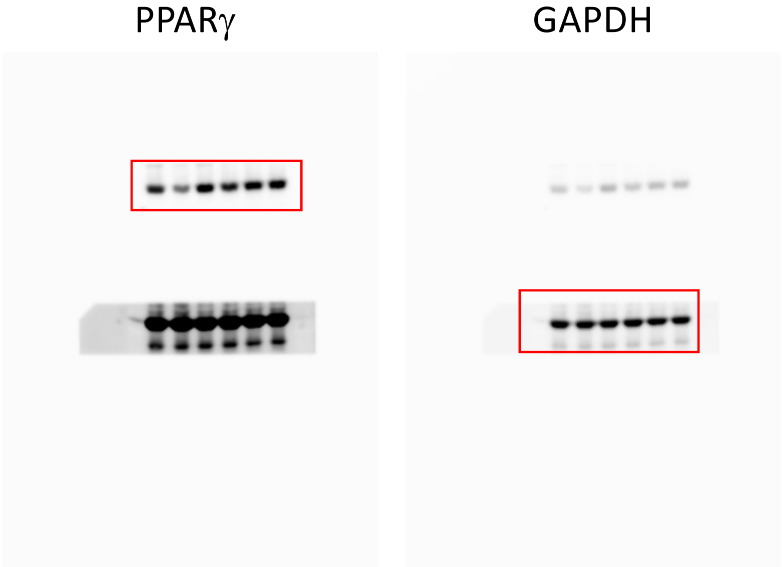

Fig. 5D

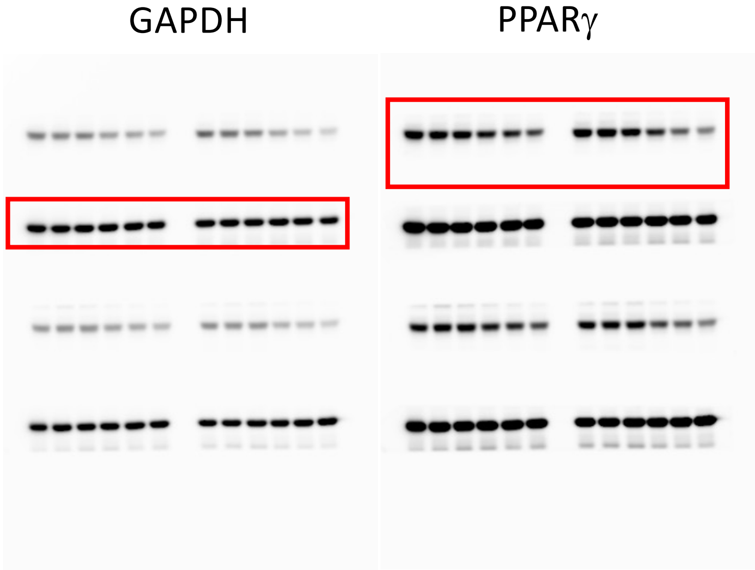

Fig. 6A

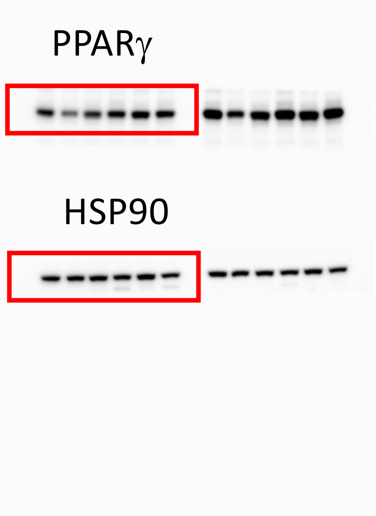

Fig. 6B

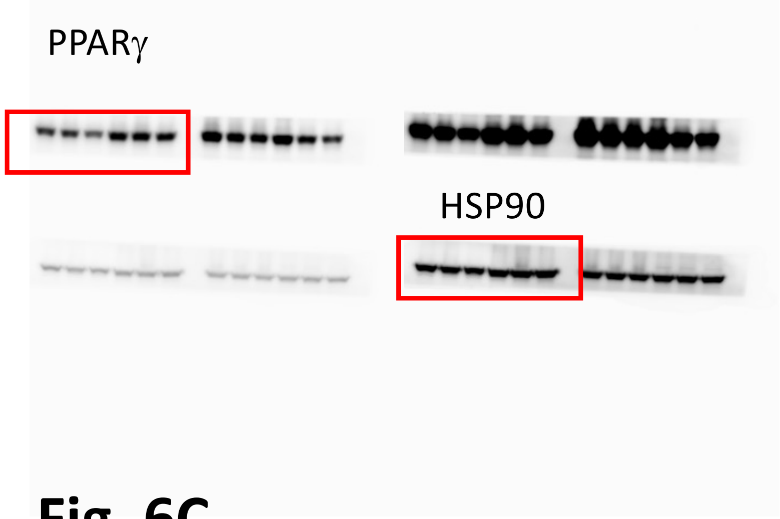

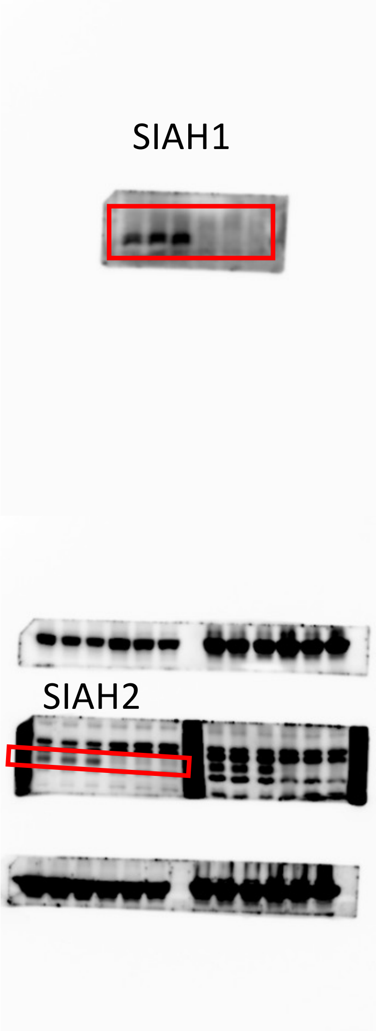

Fig. 6C

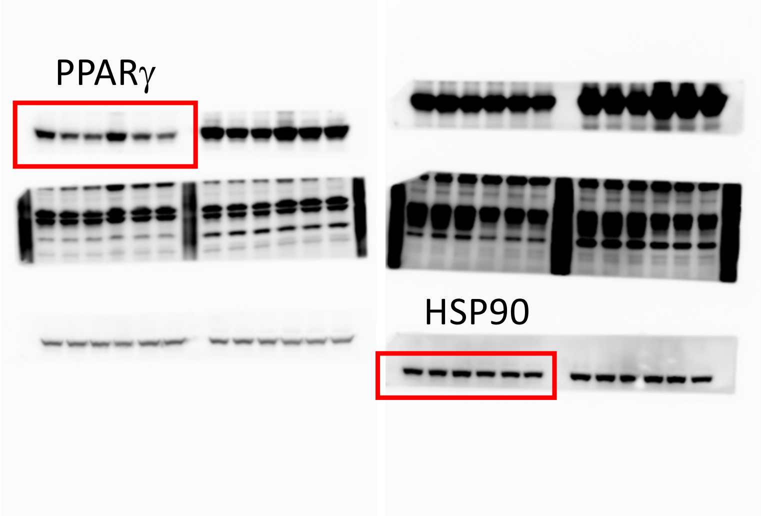

Fig. 6D

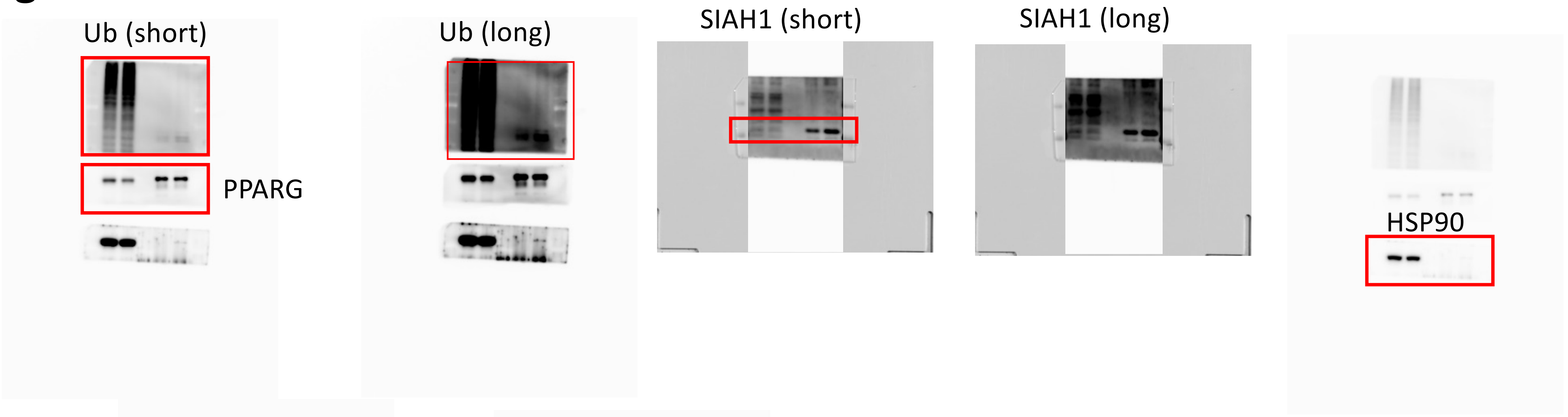
Ub (short)
Ub (long)
SIAH1 (short)
SIAH1 (long)
PPARG
HSP90

Fig. 6F

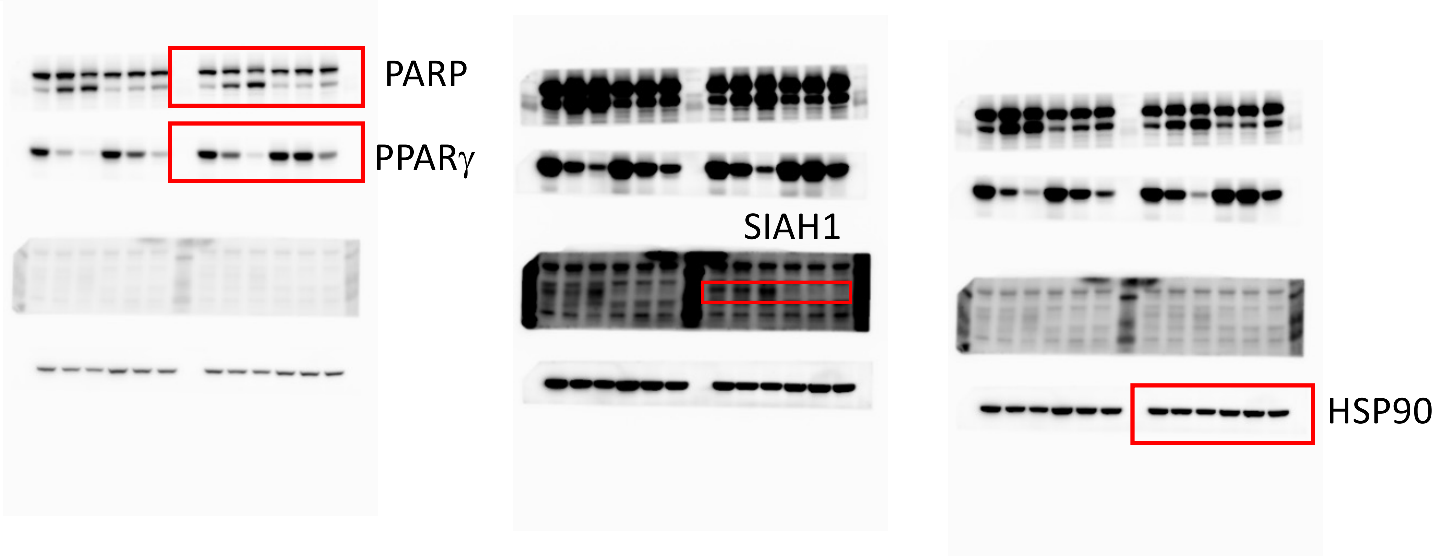
PARP
PPARγ
SIAH1
HSP90

Fig. 6F (continued)

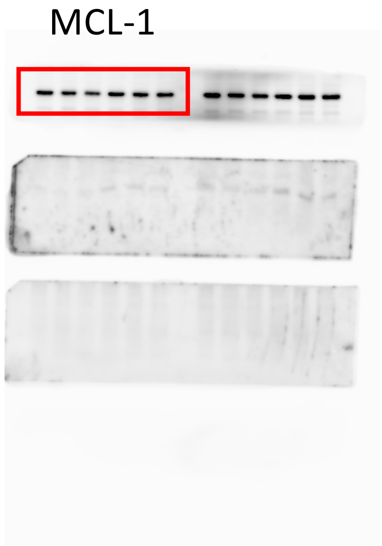

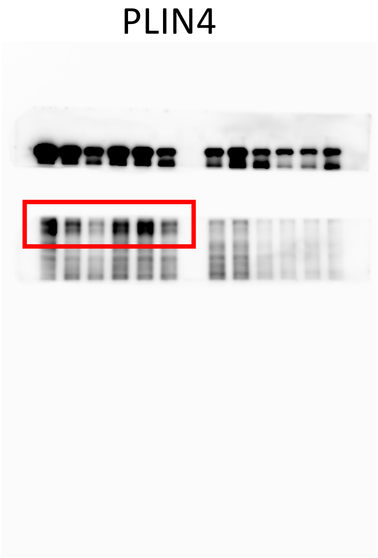

Fig.S1 D

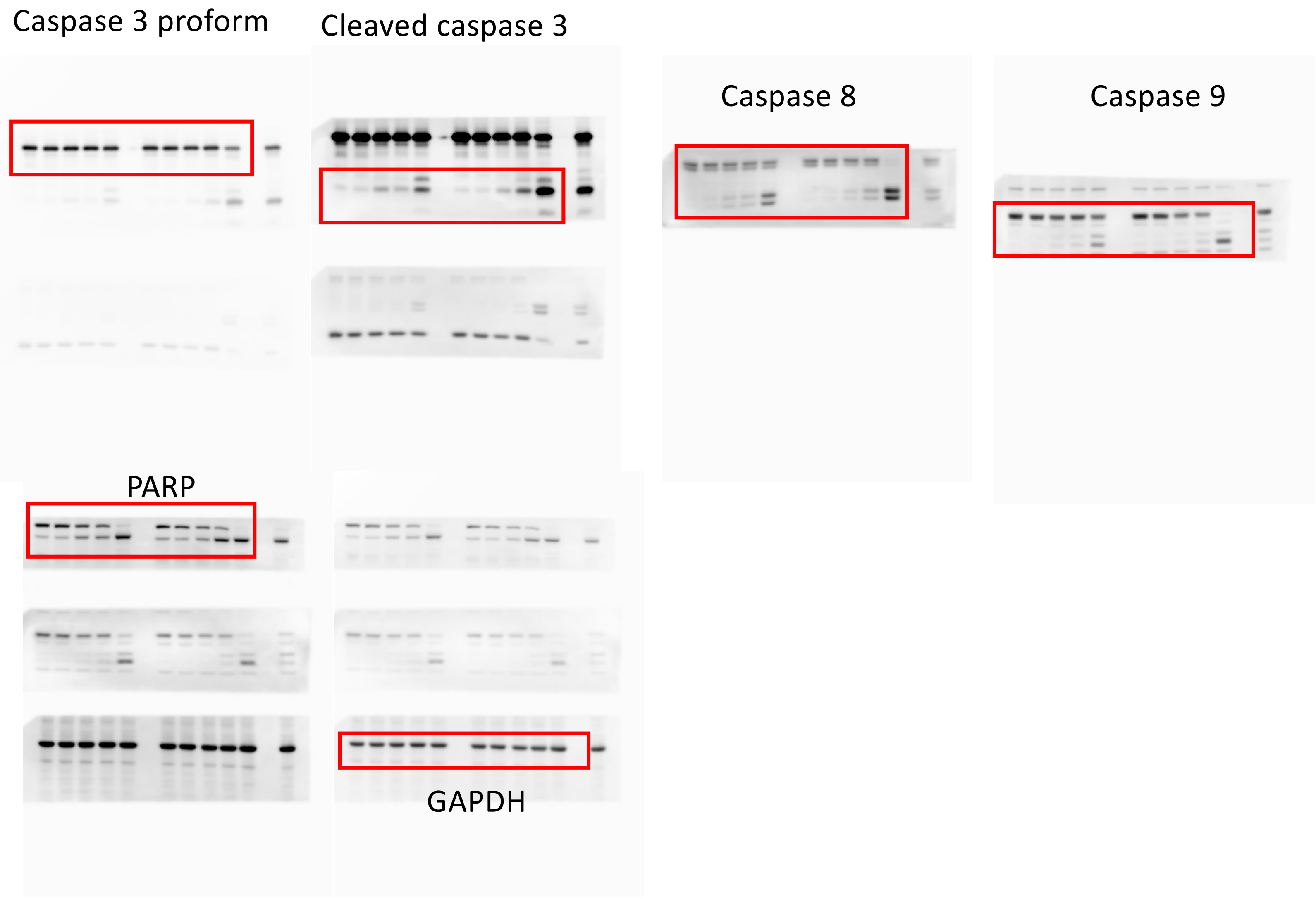
Caspase 3 proform
Cleaved caspase 3
Caspase 8
Caspase 9
PARP
GAPDH

Fig. S3E

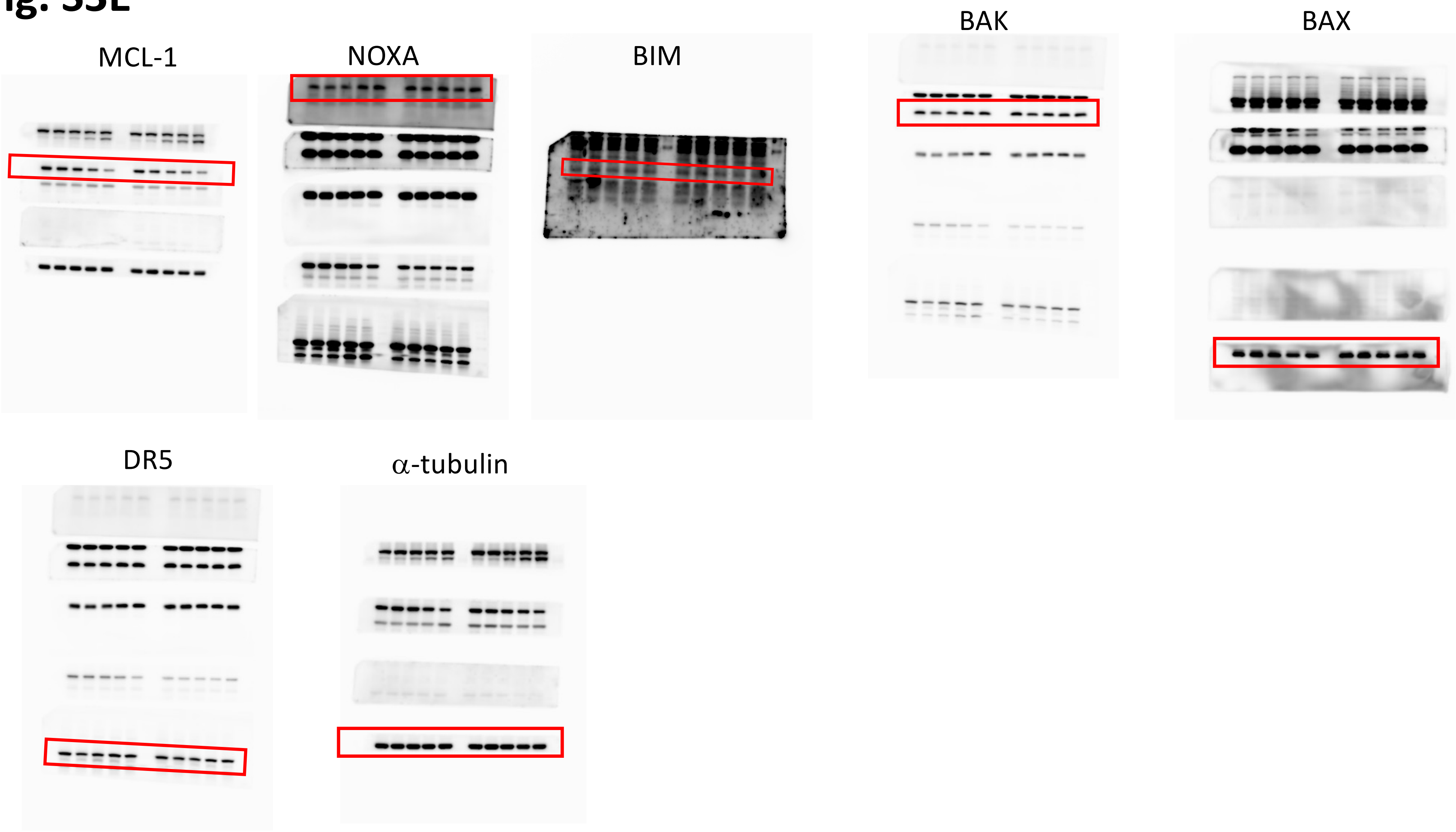

Fig. S4C, D, E

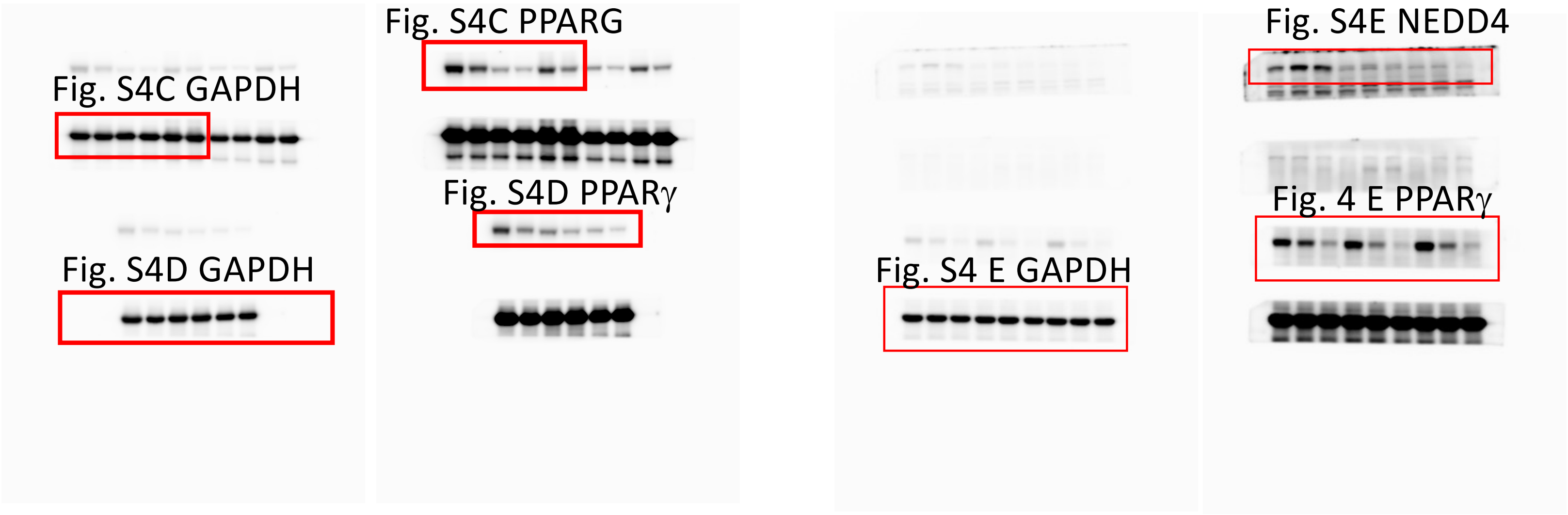

Fig. S4C GAPDH
Fig. S4D GAPDH
Fig. S4C PPARG
Fig. S4D PPARγ
Fig. S4 E GAPDH
Fig. S4E NEDD4
Fig. 4 E PPARγ

Fig. S5A

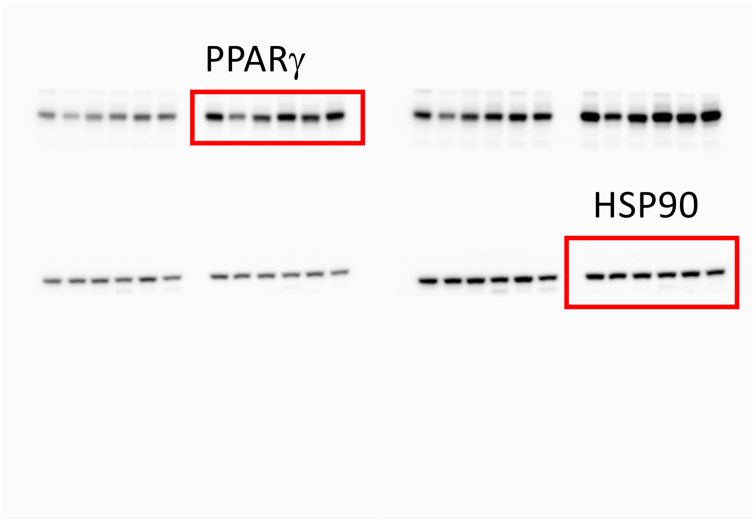

Fig. S5C

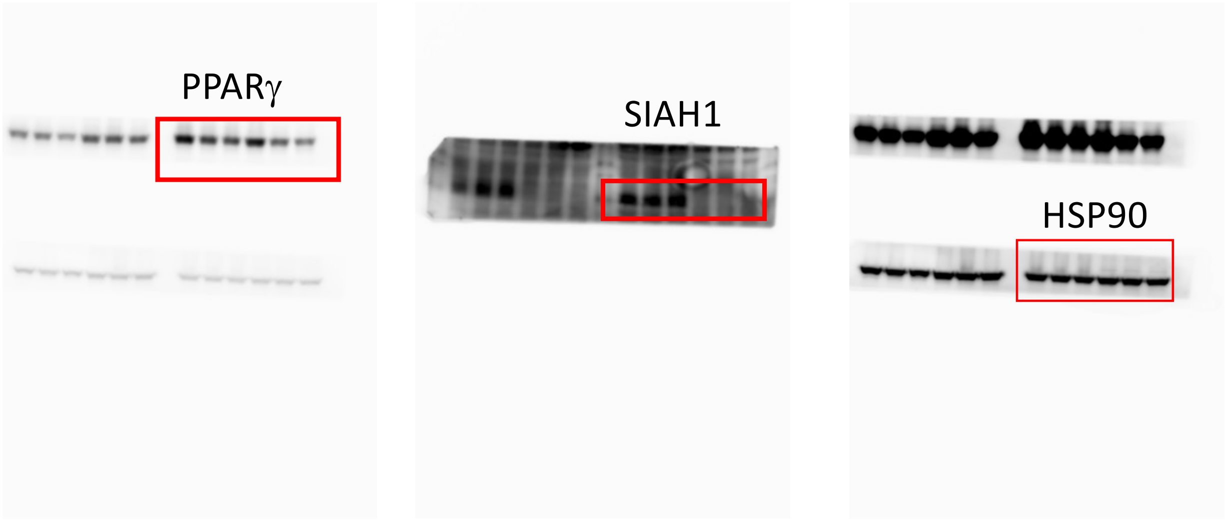

Fig. S5D

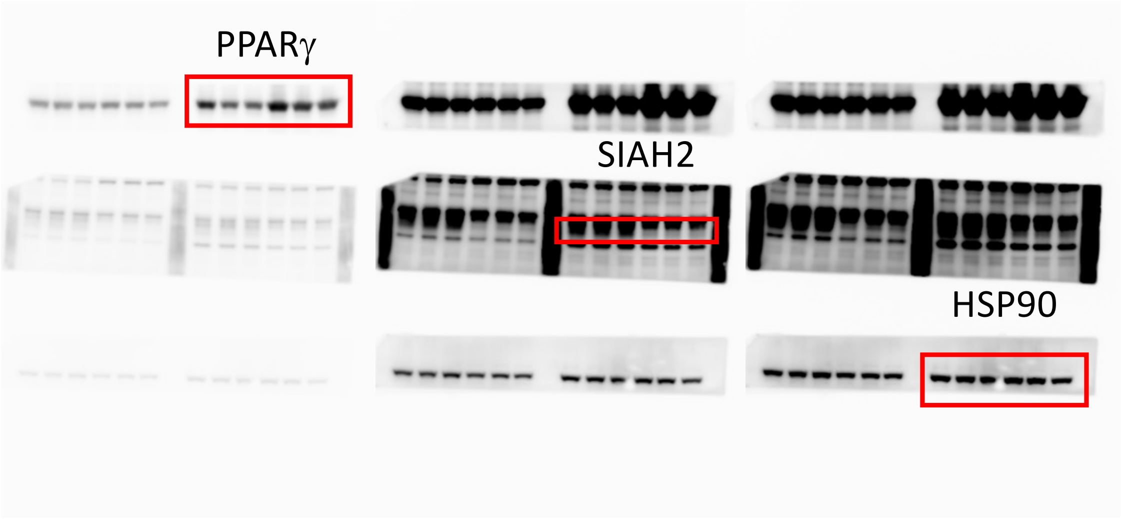

Fig. S5B

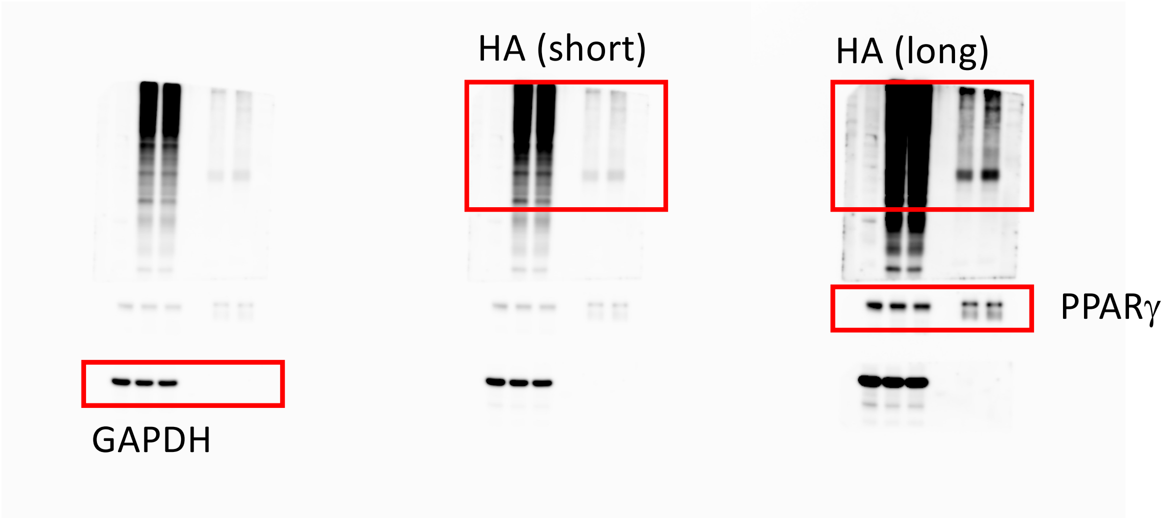

Fig. S5E

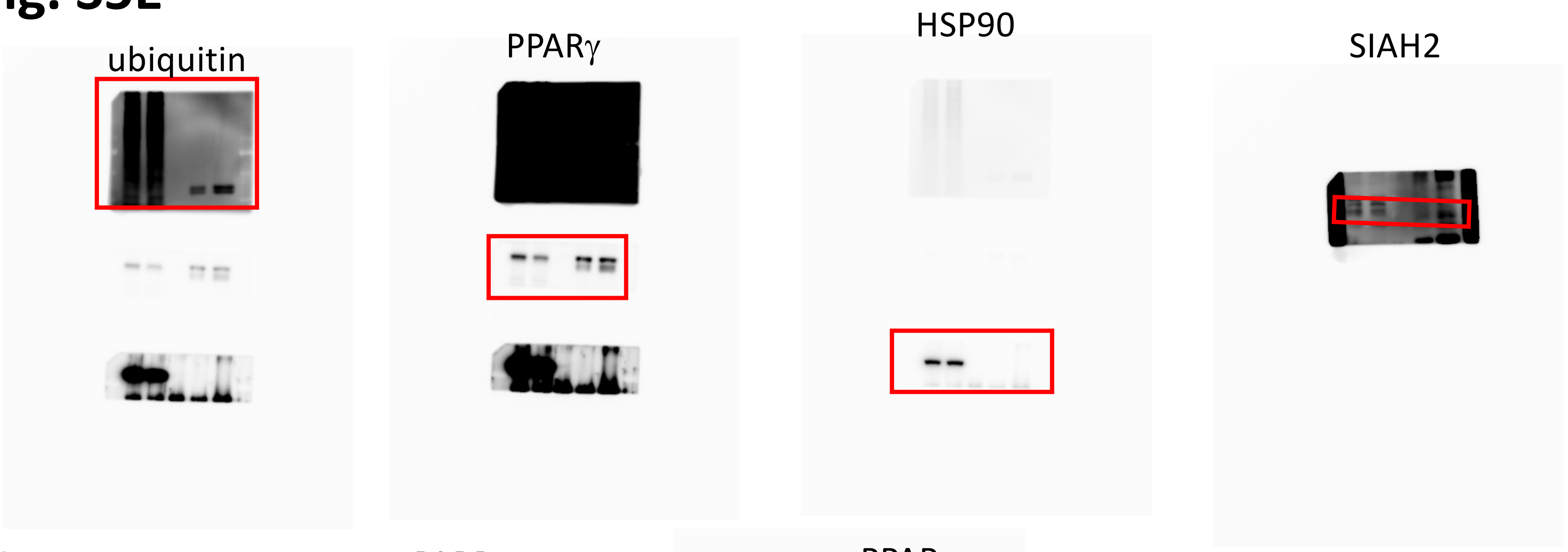

Fig. S4F

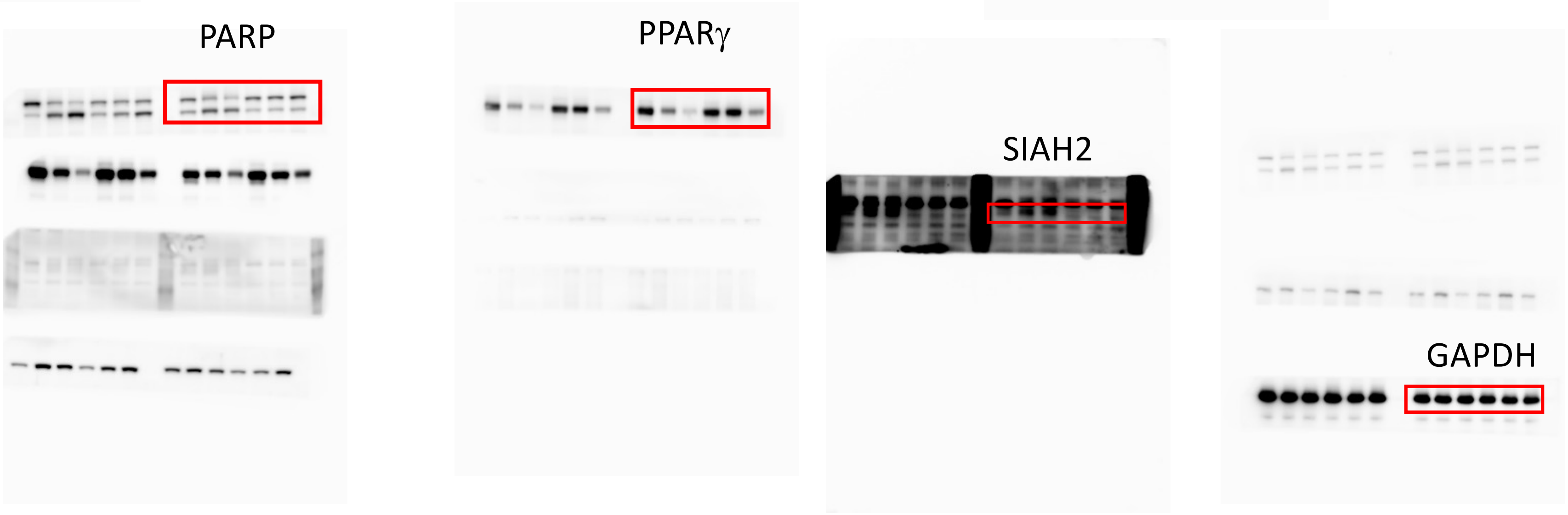

Original data of RT-qPCR

Fig. 3D

PPAR-γ Levels				
Cinobufotalin (μM)	CTRL	0.31	0.625	1.25
N=1	1.00	1.34	0.73	0.98
N=2	1.00	0.64	0.67	0.83
Mean	1.00	0.99	0.70	0.91
SD	0	0.49	0.04	0.11
T test		0.9835	0.0093	0.3524

PLIN4 Levels				
Cinobufotalin conc.	CTRL	0.31	0.625	1.25
N=1	1.00	0.86	0.46	0.14
N=2	1.00	0.36	0.26	0.13
Mean	1.00	0.61	0.36	0.13
SD	0	0.36	0.14	0.01
T test		0.2627	0.0228	0.00003

HMGCS2 Levels				
Cinobufotalin (μM)	CTRL	0.31	0.625	1.25
N=1	1.00	0.04	0.04	0.07
N=2	1.00	0.05	0.06	0.13
Mean	1.00	0.04	0.05	0.10
SD	0	0.002	0.01	0.04
T test		0.000002	0.00011	0.00094

FASN Levels				
Cinobufotalin conc.	CTRL	0.31	0.625	1.25
N=1	1.00	0.62	0.40	0.52
N=2	1.00	0.44	0.41	0.45
Mean	1.00	0.53	0.41	0.49
SD	0	0.13	0.01	0.05
T test		0.0357	0.0002	0.0050

EHHADH Levels				
Cinobufotalin (μM)	CTRL	0.31	0.625	1.25
N=1	1.00	0.16	0.02	0.00
N=2	1.00	0.10	0.03	0.10
Mean	1.00	0.13	0.03	0.05
SD	0	0.04	0.002	0.07
T test		0.0011	0.000003	0.0027

Fig. 3G

PPAR-γ Levels							
Cell lines	SV-HUC-1	RT112	RT4	T24	J82	UM-UC-3	5637
N=1	1.00	1.89	4.94	1.24	1.84	0.21	4.84
N=2	1.00	3.18	5.36	1.10	0.93	0.04	2.48
N=3	1.00	2.73	5.99	0.68	1.05	0.02	2.85
Mean	1.00	2.54	5.15	1.17	1.38	0.13	3.66
SD	0	0.65	0.52	0.29	0.50	0.11	1.27
T test		0.0065	0.00006	0.4881	0.1960	0.00006	0.0156

FASN Levels							
Cell lines	SV-HUC-1	RT112	RT4	T24	J82	UM-UC-3	5637
N=1	1.00	2.17	1.25	0.92	1.09	0.78	1.32
N=2	1.00	3.26	1.47	0.70	0.51	0.16	0.59
N=3	1.00	2.64	1.31	0.94	0.88	0.55	1.43
Mean	1.00	2.72	1.36	0.81	0.80	0.47	0.95
SD	0	0.55	0.11	0.13	0.29	0.31	0.46
T test		0.0029	0.0031	0.0676	0.1830	0.0250	0.3467

FABP4 Levels							
Cell lines	SV-HUC-1	RT112	RT4	T24	J82	UM-UC-3	5637
N=1	1.00	9305.42	160046.99	269.63	2.69	11.16	49.96
N=2	1.00	9109.00	140833.64	1122.07	93.58	28.21	264.93
N=3	1.00	10402.35	146453.13	320.86	1.36	11.12	43.47
Mean	1.00	9207.21	150440.31	695.85	48.14	19.68	157.44
SD	0	696.97	9878.63	478.06	52.86	9.85	126.03
T test		0.00001	0.00001	0.0539	0.1799	0.0249	0.0894

PLIN4 Levels							
Cell lines	SV-HUC-1	RT112	RT4	T24	J82	UM-UC-3	5637
N=1	1.00	81.88	147.31	0.86	0.20	0.16	0.91
N=2	1.00	118.32	71.98	0.62	0.88	0.52	1.21
N=3	1.00	174.53	143.52	0.69	10.30	0.28	0.71
Mean	1.00	100.10	109.65	0.74	0.54	0.34	1.06
SD	0	46.68	42.44	0.12	5.65	0.18	0.25
T test		0.00502	0.00404	0.00867	0.21999	0.00147	0.35805
