# Supplementary material for: Targeting PPARγ via SIAH1/2-mediated ubiquitin-proteasomal degradation as a new therapeutic approach in luminal-type bladder cancer
Source: Cell Death Dis. 2024 Dec 18;15(12):908. doi: 10.1038/s41419-024-07298-x (PMC11655661; doi:10.1038/s41419-024-07298-x)
Supplement: Supplementary file 2 — Supplementary materials [file 41419_2024_7298_MOESM2_ESM.docx]

**Targeting PPARγ via SIAH1/2-Mediated Ubiquitin-Proteasomal Degradation as a New Therapeutic Approach in Luminal-Type Bladder Cancer**

**Chih-Chieh Tu^1,2^, Tsung-Han Hsieh^3^, Cheng-Ying Chu^4,5^, Yu-Chen Lin^1,2^, Bo-Jyun Lin^1,2^, Chun-Han Chen^1,2,6 *^**

^1^ Department of Pharmacology, School of Medicine, College of Medicine, Taipei Medical University, Taipei 110, Taiwan.

^2^ Graduate Institute of Medical Sciences, College of Medicine, Taipei Medical University, Taipei 110, Taiwan.

^3^ Precision Health Center, Taipei Medical University, Taipei 110, Taiwan.

^4^ CRISPR Gene Targeting Core, Taipei Medical University, Taipei 110, Taiwan

^5^ TMU Research Center of Cancer Translational Medicine, Taipei Medical University, Taipei 110, Taiwan

^6^ Cell Physiology and Molecular Image Research Center, Wan Fang Hospital, Taipei Medical University, Taipei 116, Taiwan.

**Running title:** SIAH1/2-mediated proteasomal degradation of PPARγ

**^*^Corresponding author:**

Chun-Han Chen, Ph.D.

Associate Professor, Department of Pharmacology, School of Medicine, College of Medicine, Taipei Medical University, Taipei 110, Taiwan.

E-mail address: [brianchc@tmu.edu.tw](mailto:brianchc@tmu.edu.tw)

**Supplementary Figures**

**
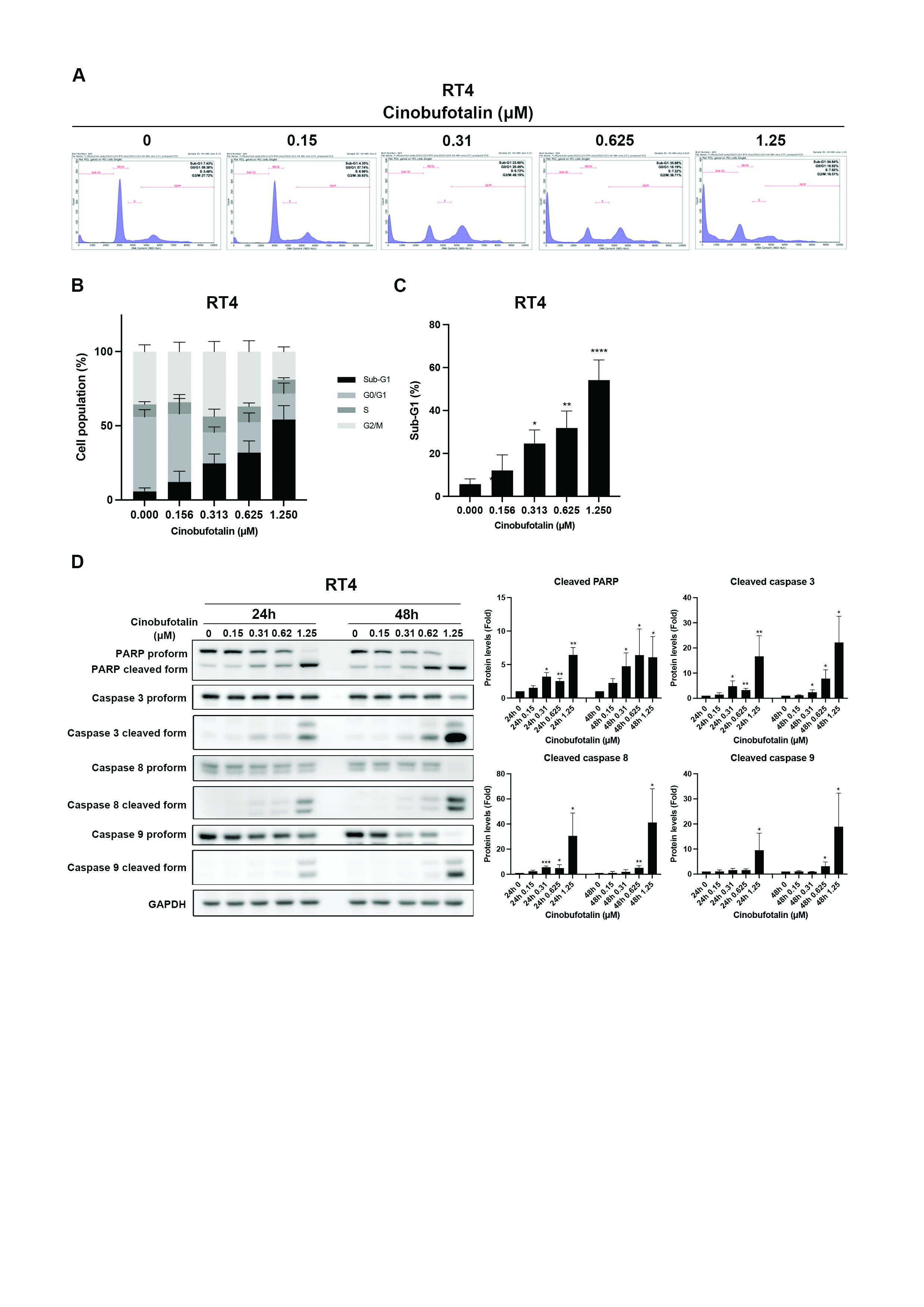
**

**Supplementary Figure S1. Cinobufotalin induces cell-cycle accumulation at sub-G1 phase and apoptosis in RT4 cells. A–C** Effect of cinobufotalin on cell-cycle distribution in RT4 cells. Cells were treated with different concentrations of cinobufotalin for 48h, and then were stained by propidium iodide, and cell cycle was analyzed by flow cytometry (**A**). Quantitative data of histogram (**B–C**) are expressed as mean±S.D. (n=3) **p*<0.05, ***p*<0.01 and *****p*<0.0001 compared with control group. **D** Cinobufotalin activates apoptotic proteins in RT4 cells. Cells were treated by indicated concentrations of cinobufotalin for 24 h and 48 h, and protein expression was analyzed by western blot. Band intensities of each protein were quantified by Image J, and normalized to GAPDH. Fold changes compared to control group were depicted, and data are expressed as mean±S.D. (n=3). **p*<0.05, ***p*<0.01 and ****p*<0.001 compared with control group.


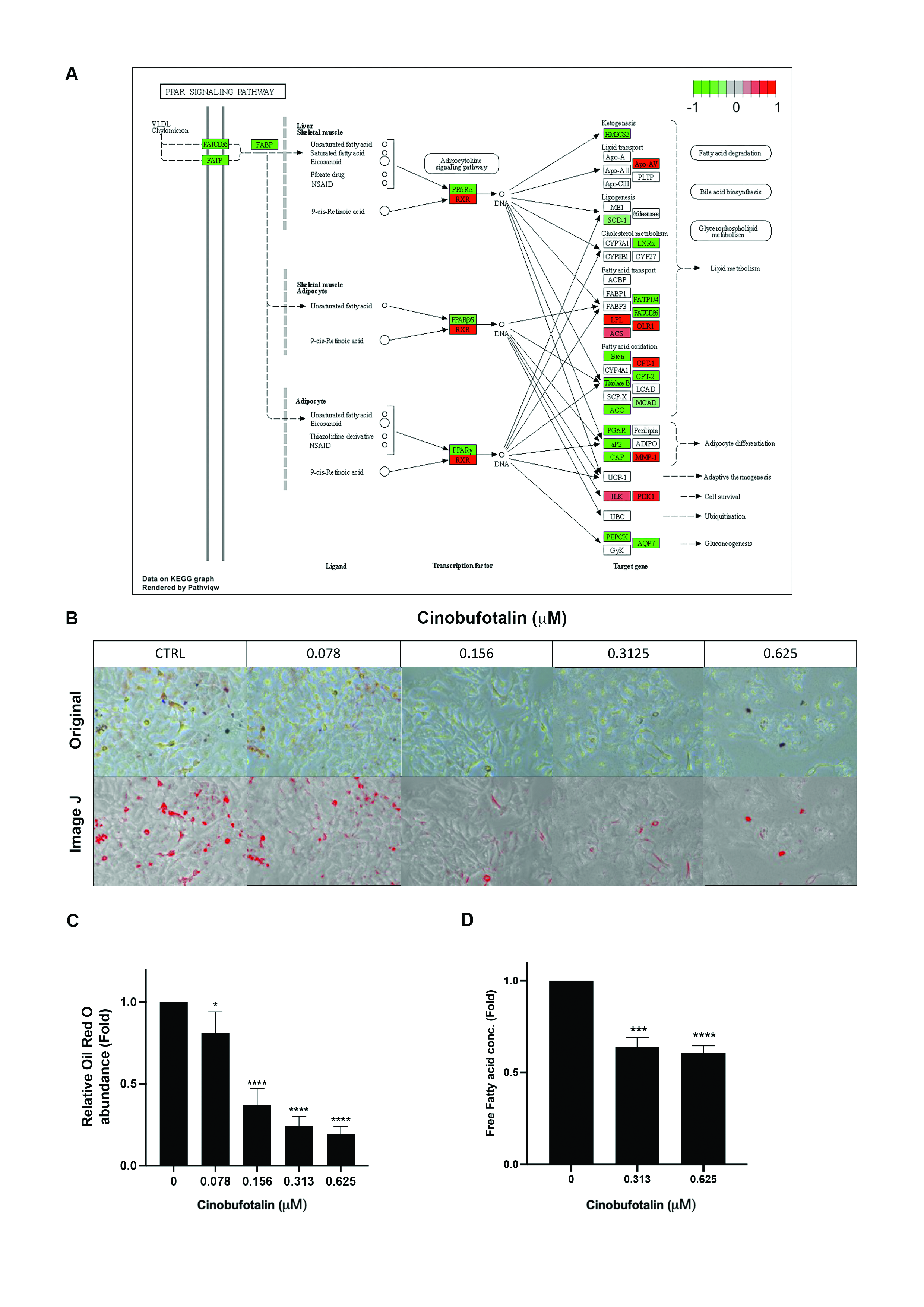


**Supplementary Figure S2. Cinobufotalin decreases the levels of lipid droplets and free fatty acid in RT112 cells.** **A** RT112 cells were treated with cinobufotalin (0.625 μM) for 24 h. RNA was extracted using TRIzol reagent, followed by next-generation sequencing and bioinformatics analyses (n=2). The PPAR signaling pathway is significantly enriched and the up- (red) and downregulated (green) genes are depicted. **B–C** The effect of cinobufotalin on lipid droplet formation. Cells were treated with cinobufotalin for 24 h and subjected to Oil Red O staining (**B**). The relative Oil red O-positive areas were analyzed with the Image J software (**C**). Data are expressed as mean±S.D. (n=3) **p*<0.05 and *****p*<0.0001 compared with control. **D** Cells were exposed to the indicated concentrations of cinobufotalin for 24 h and the free fatty acid levels were determined. Data are expressed as mean±S.D. (n=3) ****p*<0.001 and *****p*<0.0001 compared with control.


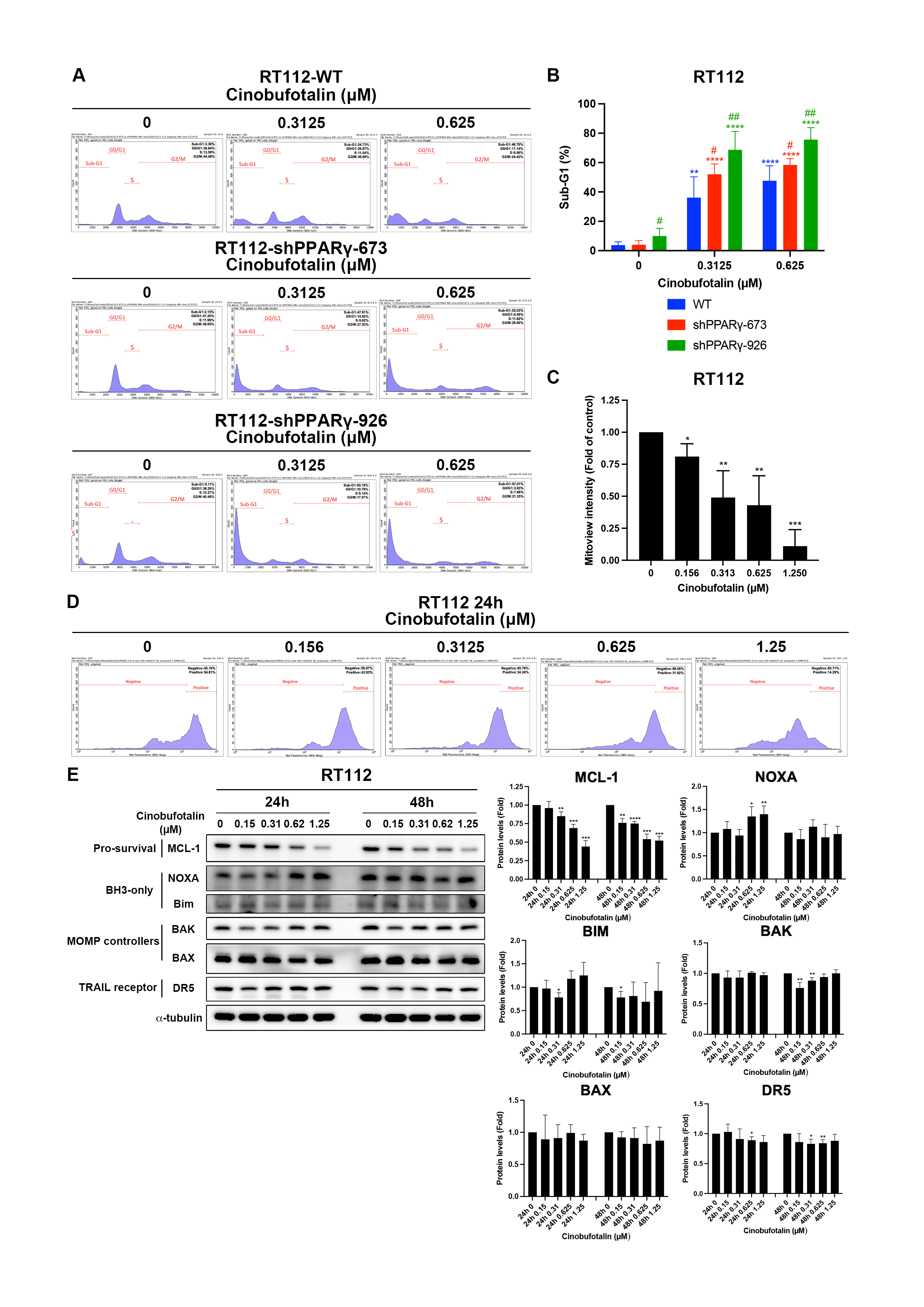


**Supplementary Figure S3. PPARγ knockdown potentiates cinobufotalin-induced apoptosis in RT112 cells. A–B** PPARγ was stably knocked down using two different shRNA sequences (673 and 926), followed by treatment with indicated concentrations of cinobufotalin for 48 h. The cells were stained with propidium iodide (PI), and cell cycle distribution was analyzed using flow cytometry (**A**). Quantitative data from the histograms are presented as the mean±S.D. (n=4). ***p*<0.01 and *****p*<0.0001 compared to the control group; **^#^***p*<0.05 and **^##^***p*<0.01 compared to the wild type (WT) group (**B**). **C–D** Effect of cinobufotalin on mitochondrial membrane potential in RT112 cells. After the treatment of different concentrations of cinobufotalin for 24 h, the cells were stained by 100 nM MitoView^TM^ 633 dye for 15 minutes, and mitochondrial membrane potential was then analyzed by using flow cytometry (**D**). Quantitative data are presented as mean±S.D. (n=3). **p*<0.05, ***p*<0.01 and ****p*<0.001 compared to the control group (**C**). **E** The effects of cinobufotalin on apoptosis regulatory proteins in RT112 cells. The cells were treated with cinobufotalin for 24 h or 48 h, and subjected to western blotting by indicated antibodies. Band intensities of each protein were quantified by Image J and normalized to α-tubulin. Fold changes relative to the control group are shown and presented as mean±S.D (n=3). **p*<0.05, ***p*<0.01, ****p*<0.001 and ****p<0.0001 compared to the control group.


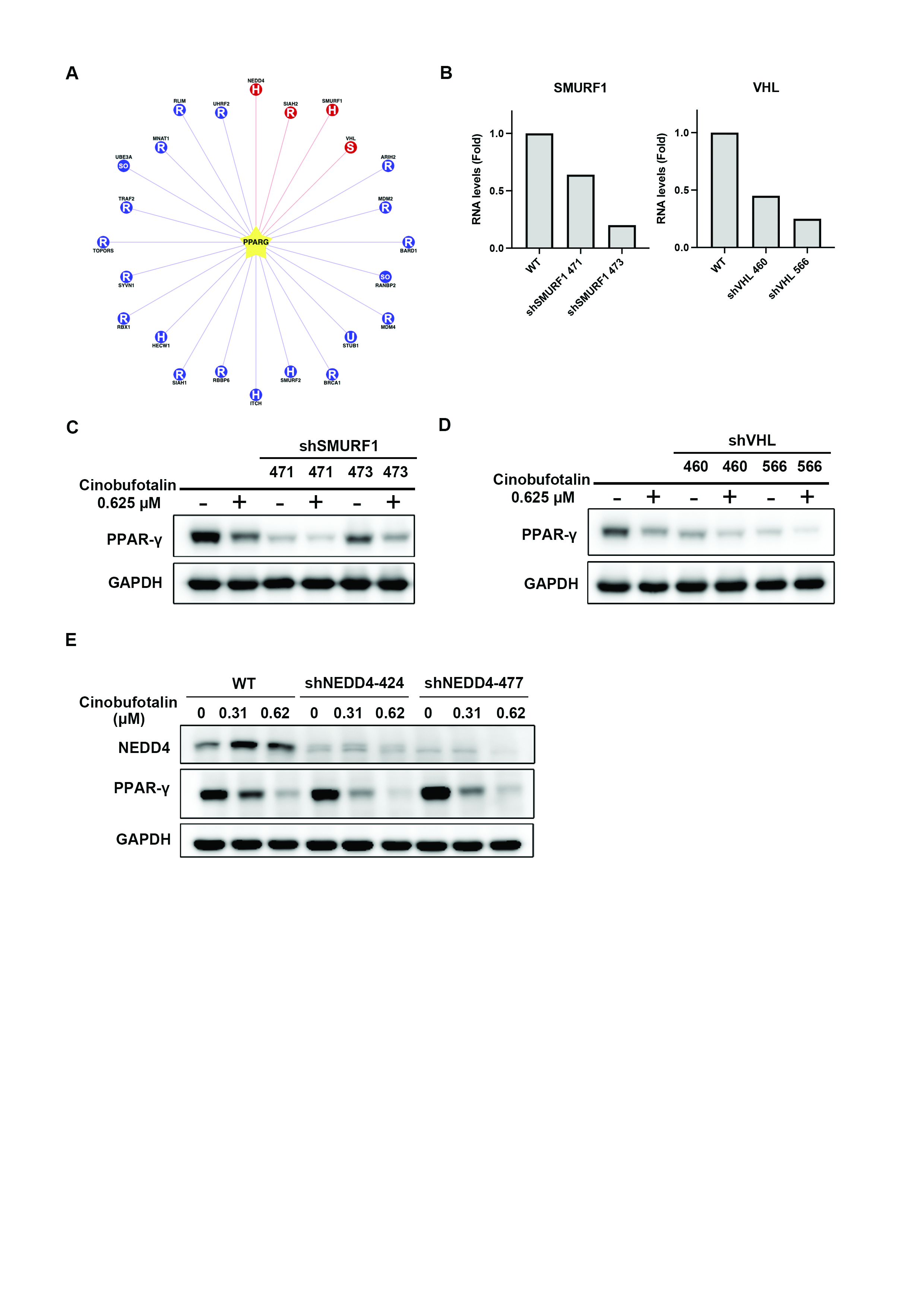


**Supplementary Figure S4. Exploring potential E3 ubiquitin ligases regulating cinobufotalin-induced proteasomal degradation of PPARγ in RT112 cells. A** The network view of the predicted E3 ubiquitin ligase of PPARγ by UbiBrowser 2.0 (H HECT; R RING; S SOCS; SO OTHER; U UBOX). PPARγ locates in the center of the canvas, and the predicted E3 ligases surround the substrate. **B–E** RT112 cells were transduced with lentivirus carrying indicated shRNA, and knockdown efficiency was evaluated by qPCR (**B**) or Western blot (**E**). RT112 cells with or without knockdown of E3 ubiquitin ligase were exposed to cinobufotalin for 3 h, and subjected to Western blot analysis (**C–E**).


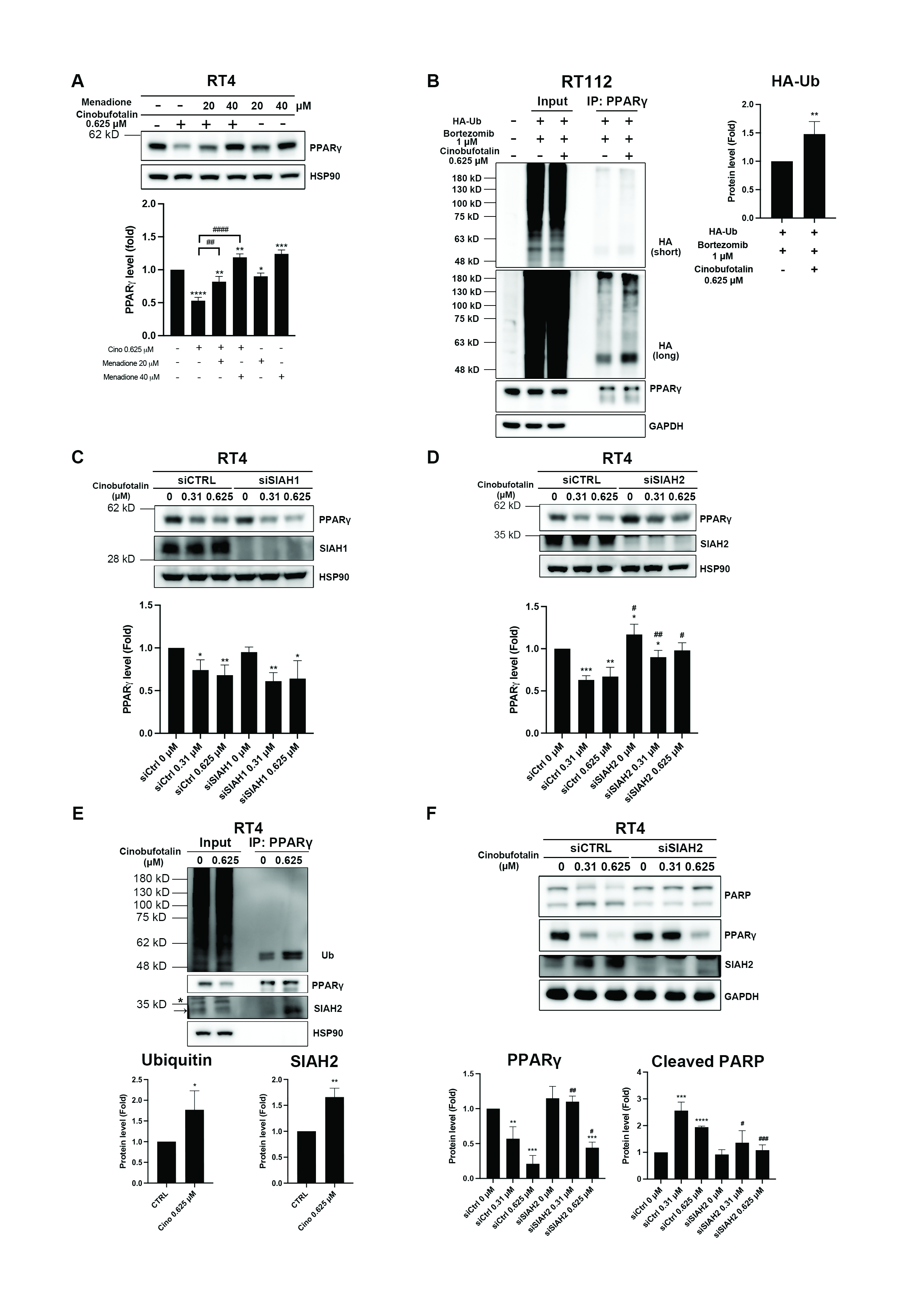


**Supplementary Figure S5. Identifying E3 ubiquitin ligase in mediating cinobufotalin-induced proteasomal degradation of PPARγ in luminal BC cells. A** SIAH1/2 inhibitor reversed cinobufotalin-induced degradation of PPARγ in RT4 cells. The cells were pre-incubated with menadione for 30 min, and then treated with cinobufotalin for 3 h. The cells were subjected to western blot analysis, and the band intensities of each protein were determined by using the Image J software. Data are expressed as mean±S.D. (n=3) **p*<0.05, ***p*<0.01, ****p*<0.001 and *****p*<0.0001 compared with control; **^##^***p*<0.01 and **^####^***p*<0.0001 compared with cinobufotalin 0.625 μM alone. **B** RT112 cells were transiently transfected with HA-Ub plasmid, and pretreated with bortezomib (1 μM) for 1 h, followed by exposing to cinobufotalin (0.625 μM) for 2 h. Cells were then subjected to immunoprecipitation using an anti-PPARγ antibody, and the precipitated proteins were analyzed by western blot. Band intensities of each protein were determined by using the Image J software. Data are expressed as mean±S.D. (n=3) ***p*<0.01 compared to the control group. **C–E** SIAH2 contributes significantly to the cinobufotalin-induced ubiquitin-proteasomal degradation of PPARγ in RT4 cells. **C–D** RT4 cells were transfected with siRNA targeting SIAH1 (**C**) or SIAH2 (**D**), and exposed to indicated concentrations of cinobufotalin for 3 h. The cells were subjected to western blot analysis, and the band intensities of each protein were determined by using the Image J software. Data are expressed as mean±S.D. (n=3) **p*<0.05, ***p*<0.01 and ****p*<0.001, compared with control; **^#^***p*<0.05 and **^##^***p*<0.01 compared with siCTRL group. (**E**) RT4 cells were exposed to cinobufotalin (0.625 μM) for 3 h, and subjected to immunoprecipitation analysis by using antibody against PPARγ followed by Western blotting. Band intensities of each protein were determined by using the Image J software. Data are expressed as mean±S.D. (n=3) **p*<0.05 and ***p*<0.01 compared with control (CTRL). **F** RT4 cells were transiently transfected with non-targeting control (siCTRL) or siRNA targeting SIAH2. After transfection, the cells were exposed to cinobufotalin for 48 h, and subjected to Western blot analysis. Band intensities of each protein were determined by using the Image J software and normalized to GAPDH. Data are expressed as mean±S.D. ***p*<0.01, ****p*<0.001 and *****p*<0.0001 compared with control group; **^#^***p*<0.05, **^##^***p*<0.01 and **^###^***p*<0.001 compared with siCTRL group.


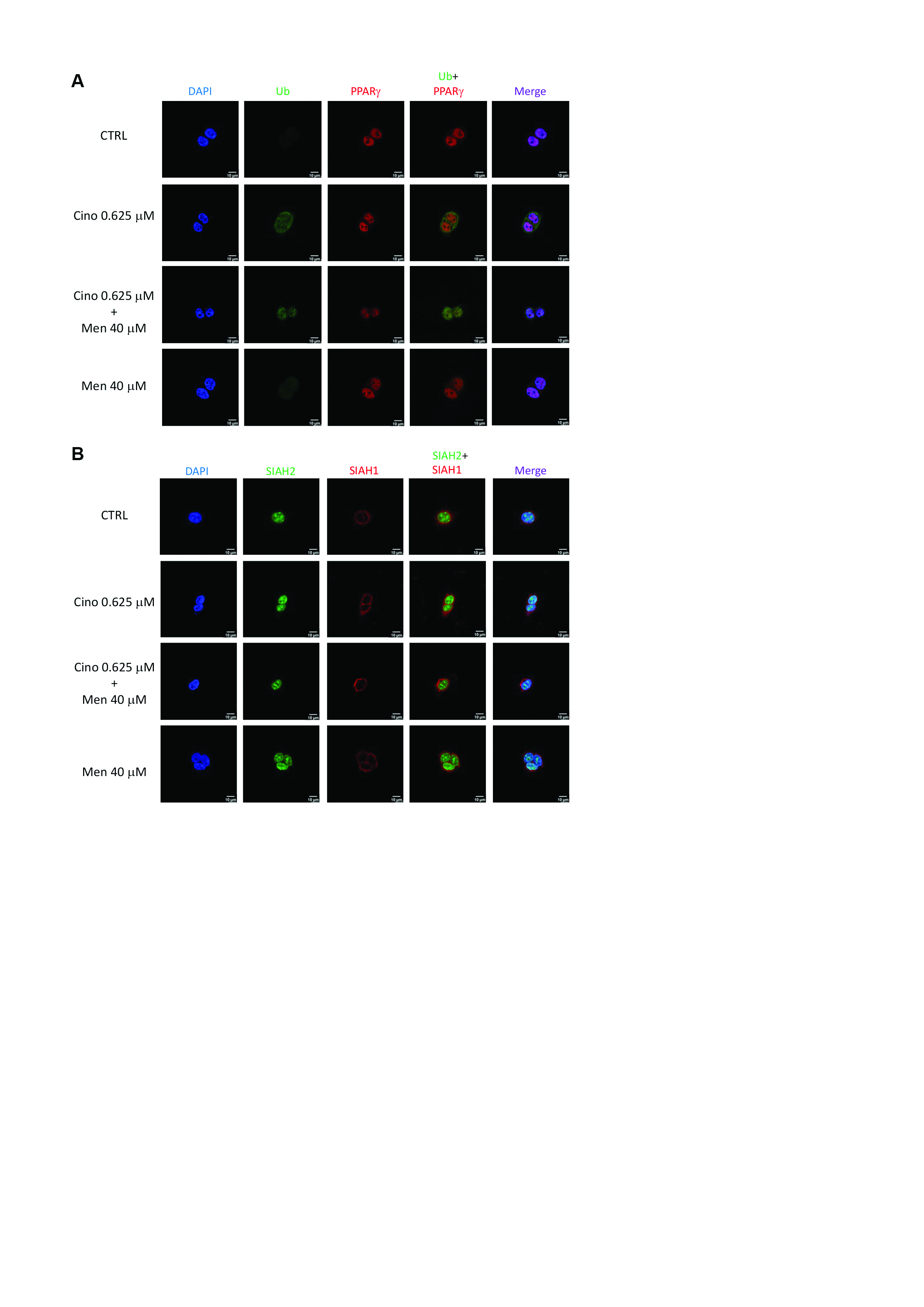


**Supplementary Figure S6. Nuclear distribution of SIAH2 is crucial to cinobufotalin-induced ubiquitination of PPARγ in RT4 cells.** **A–B** RT4 cells were treated with cinobufotalin (0.625 μM) in the presence or absence of menadione (40 μM) for 3 h and subjected to immunofluorescence staining with antibodies against ubiquitin (Ub) and PPARγ (**A**) or SIAH1 and SIAH2 (**B**).

**
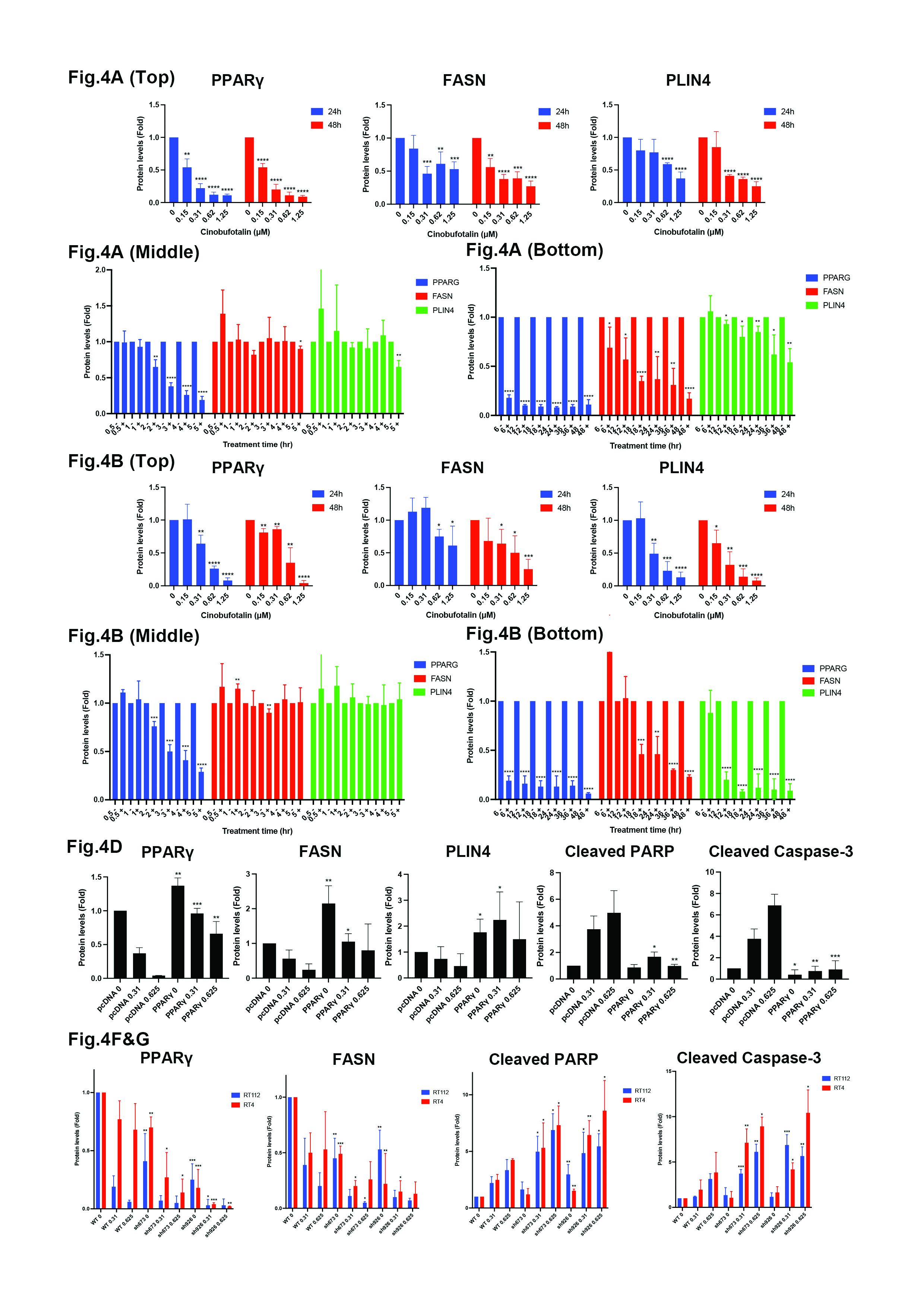
**

**Supplementary Figure S7. The statistical analysis of western blots in Figure 4.**

The band intensities of each protein in Fig. 4A and Fig. 4B were quantified by Image J, and normalized to GAPDH. Fold changes compared to control group were depicted. Data are expressed as mean±S.D (n=3). **p*<0.05, ***p*<0.01, ****p*<0.001 and *****p*<0.0001 compared with control group. **D** Band intensities of each protein in Fig. 4D were determined by using the Image J software. After normalizing with the intensity of GAPDH, relative protein level was determined by comparing with control group. Data are expressed as mean±S.D (n=3). **p*<0.05, ***p*<0.01 and ****p*<0.001 compared with pcDNA group. The band intensities of each protein in Fig. 4F and Fig. 4G were determined by using the Image J software. After normalizing with the intensity of GAPDH, relative protein level was compared with control group. Data are expressed as mean±S.D (n=3). **p*<0.05, ***p*<0.01 and ****p*<0.001 compared with WT group.

**Supplementary Tables**

**Supplementary Table S1. Antibody information of this study**

| Primary antibodies | | | |
| --- | --- | --- | --- |
| Protein name | Catalog number | Company | Application |
| α-tubulin | GTX112141 | GeneTex | WB |
| BAK | sc-832 | Santa Cruz Biotechnology | WB |
| BAX | sc-7480 | Santa Cruz Biotechnology | WB |
| BIM | sc-11425 | Santa Cruz Biotechnology | WB |
| Caspase-3 | NB100-56708 | Novus Biologicals | WB |
| Caspase-8 | 9746 | Cell SignalingTechnology | WB |
| Caspase-9 | 9502 | Cell SignalingTechnology | WB |
| DR5 | ab8416 | Abcam | WB |
| FABP4 | 12802-1-AP | Proteintech | WB |
| FASN | 3180 | Cell SignalingTechnology | WB |
| FLAG M2 | F3165 | Thermo Fisher Scientific | WB |
| GAPDH | GTX100118 | GeneTex | WB |
| HA | GTX628489 | GeneTex | WB |
| HSP90 | sc-7947 | Santa Cruz Biotechnology | WB |
| MCL1 | sc-819 | Santa Cruz Biotechnology | WB |
| NEDD4 | 21698-1-AP | Proteintech | WB |
| NOXA | sc-30209 | Santa Cruz Biotechnology | WB |
| PARP | 9542 | Cell SignalingTechnology | WB |
| PPARγ | 2435 | Cell SignalingTechnology | WB,IF,IP |
| PPARγ | sc-7273 | Santa Cruz Biotechnology | WB |
| PLIN4 | HPA044682 | Sigma-Aldrich | WB |
| SIAH1 | 13886-1-AP | Proteintech | WB |
| SIAH2 | 12651-1-AP | Proteintech | WB |
| Ubiquitin | sc-8017 | Santa Cruz Biotechnology | WB,IF |
| Secondary antibodies | | | |
| Protein name | Catalog number | Company | Application |
| Anti-Mouse IgG | 115-035-003 | Jackson ImmunoResearch Laboratories | WB |
| Anti-Rabbit IgG | 111-035-003 | Jackson ImmunoResearch Laboratories | WB |
| Drop-n-Stain™ CF^®^488 Dye Goat Anti-Mouse IgG (H+L) | 20956 | Biotium | IF |
| Drop-n-Stain™ CF^®^594A Dye Goat Anti-Rabbit IgG (H+L) | 20955 | Biotium | IF |

Abbreviation: WB (western blot), IF (immunofluorescence), IP (immunoprecipitation)

**Supplementary Table S2. The information of shRNA**

| **Gene** | **shRNA number** | **Target sequence** |
| --- | --- | --- |
| PPARγ | TRCN0000001673 | CAGCATTTCTACTCCACATTA |
| PPARγ | TRCN0000355926 | ATGGAGTCCACGAGATCATTT |
| SMURF1 | TRCN0000003471 | GCCCAGAGATACGAAAGAGAT |
| SMURF1 | TRCN0000003473 | CTGGAGGTTTATGAGAGGAAT |
| VHL | TRCN0000010460 | TATCACACTGCCAGTGTATAC |
| VHL | TRCN0000344566 | TATCACACTGCCAGTGTATAC |
| NEDD4 | TRCN0000272424 | CGGTTGGAGAATGTAGCAATA |
| NEDD4 | TRCN0000272477 | TACGTGAGAGTGACGTTATAT |

**Supplementary Table S3. The primer sequences of real-time PCR**

| **Gene** | **Forward Primer** | **Reverse Primer** |
| --- | --- | --- |
| PPARγ | AGCCTGCGAAAGCCTTTTGGTG | GGCTTCACATTCAGCAAACCTGG |
| HMGCS2 | AAGTCTCTGGCTCGCCTGATGT | TCCAGGTCCTTGTTGGTGTAGG |
| PLIN4 | CCCACGCTTGGCAATGCTGCA | TGTTCCGCCGACAGCACCTTTG |
| EHHADH | CGGAGCATCGTGGAAAACAGCA | CCGAGTCTACAGCAATCACAGG |
| FABP4 | ACGAGAGGATGATAAACTGGTGG | GCGAACTTCAGTCCAGGTCAAC |
| FASN | TTCTACGGCTCCACGCTCTTCC | GAAGAGTCTTCGTCAGCCAGGA |
| SMURF1 | AGATCCGTCTGACAGTGTTATGT | CCCATCCACGACAATCTTTGC |
| VHL | GGAGCCTAGTCAAGCCTGAGA | CATCCGTTGATGTGCAATGCG |
| 18sRNA | CGGCGACGACCCATTCGAAC | GAATCGAACCCTGATTCCCCGTC |
